# Analysis of Intraoperative and Postoperative Hinge Fractures of Patients With Genu Valgum Treated With Lateral Open Wedge Distal Femoral Osteotomy

**DOI:** 10.1111/os.70142

**Published:** 2025-08-05

**Authors:** Zijian Lian, Bin Zhao, Jianxiong Ma, Songqing Ye, Haohao Bai, Zhihu Zhao, Xuan Jiang, Fei Xing, Yao Deng, Wei Luo, Xinlong Ma

**Affiliations:** ^1^ Department of Orthopaedics Tianjin Hospital, Tianjin University Tianjin China

**Keywords:** delayed union, genu valgum, hinge position, intraoperative hinge fracture, lateral open wedge distal femoral osteotomy, postoperative hinge fracture

## Abstract

**Objective:**

Hinge fracture is a known complication of lateral open wedge distal femoral osteotomy (LOWDFO). However, few studies have differentiated between intraoperative hinge fractures (IHF) and postoperative hinge fractures (PHF). This study aims to investigate the causes of these two types of fractures to help reduce complication rates and improve surgical outcomes.

**Methods:**

We retrospectively analyzed data from 100 patients with genu valgum deformity and lateral unicompartmental osteoarthritis who underwent distal femoral osteotomy at our hospital between January 1st, 2022, and January 1st, 2024, in our hospital. Clinical parameters, radiological data, and the associated factors influencing IHF and PHF were analyzed. Radiological data such as mechanical axis deviation (MAD) and mechanical lateral distal femur angle (mLDFA) were collected. Clinical outcomes such as osteoarthritis index and time of healing were evaluated. Based on fracture morphology, IHF and PHF were further classified into Type 1 (extension), Type 2 (distal) and Type 3 (proximal) for detailed analysis. Statistical analyses included *t*‐tests, Chi‐square tests, and regression models to identify factors associated with IHF and PHF.

**Results:**

A total of 87 patients were included in this study. The mean healing time of patients with all kinds of hinge fractures (3.4 ± 1.2 months) was longer than that of patients with no hinge fractures (2.8 ± 0.7 months), which was significant, *p* = 0.013. The MAD correction, mLDFA correction, and mLDFA correction ratio were related to hinge fractures (*p* = 0.010, 0.002, and 0.002 respectively). The body weight was higher in all types of hinge fractures group (IHF and PHF together) than the no hinge fractures group. The IHF group had a longer time of healing than the no IHF group. In the IHF group, the mLDFA correction (*p* = 0.005), mLDFA correction ratio (*p* = 0.005), and BMI (*p* = 0.031) were higher than the no IHF group. The PHF was related to hinge position. The group of hinge localized proximal to the adductor tubercle (AT) had a higher rate of PHF than the group of hinge localized in the adductor tubercle (*p* = 0.001). The healing time in the IHF group (3.9 ± 1.4 months) was significantly longer than the healing time in the PHF group (2.7 ± 0.4 months) (*p* = 0.002).

**Conclusion:**

In patients with genu valgum undergoing LOWDFO, IHF and PHF represent distinct clinical entities. IHF is associated with greater mLDFA correction, higher mLDFA correction ratios, and increased body weight. In contrast, PHF is primarily associated with hinge position, with a higher incidence observed when the hinge is located proximal to the adductor tubercle. Among the two, IHF has a more pronounced impact on delayed bone healing.

**Level of Evidence:**

Retrospective study Level IV.

## Graphical abstract

IHF and PHF are distinct complications of LOWDFO with different risk factors and clinical outcomes. Our research could reduce the risk of hinge fractures, shorten the healing time of LOWDFO, and make the treatment of genu valgum by LOWDFO more standardized and reasonable.

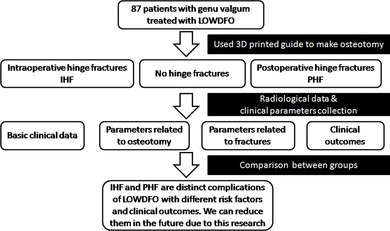

## 1 Introduction

Genu valgum is a common deformity that can affect individuals across a wide range of ages. It often results in discomfort and progressive osteoarthritis due to increased loading on the lateral compartment of the knee joint [1]. The etiology of genu valgum includes congenital disorders, trauma, degenerative changes, and limb length discrepancies [2, 3]. Distal femoral varus osteotomy has been well established as an effective treatment for valgus knee deformities [4]. Although valgus deformities are less prevalent than varus deformities, LOWDFO has emerged as a widely used surgical technique for managing genu valgum [5, 6, 7, 8]. In 2012, Takeuchi et al. introduced a classification system for hinge fractures based on the direction of the fracture line in high tibial osteotomies, distinguishing between intraoperative and postoperative hinge fractures for the first time [9]. More recently, Ogawa et al. (2024) reported that postoperative hinge fractures may be a risk factor for delayed union [10]. However, limited research has investigated hinge fractures specifically in the context of LOWDFO. Hinge fractures represent a recognized complication of LOWDFO. Yet, it remains unclear whether findings from studies on high tibial osteotomy (HTO) can be directly applied to LOWDFO? Since hinge fractures may prolong recovery time, affect postoperative function, and influence other clinical outcomes, this study was designed to fill that gap.

Recently, Winkler et al. discovered that if the hinge was distal to the adductor tubercle, the likelihood of hinge fractures in LOWDFO would be reduced [11]. While their research focused on hinge position, our study investigates the similarities and differences between IHF and PHF.

To our knowledge, this is the first study to categorize hinge fractures in LOWDFO as IHF or PHF. We sought to determine whether these two types differ in their associated risk factors and clinical outcomes. We hypothesized that IHF and PHF have distinct etiologies and impacts. By analyzing related clinical parameters and radiological data, we aimed to identify their shared and unique characteristics, which may help reduce complications such as delayed healing. Our findings may improve the acceptability of LOWDFO and inform future comparisons with open wedge high tibial osteotomy (OWHTO).

## 2 Methods

**FIGURE 1:**
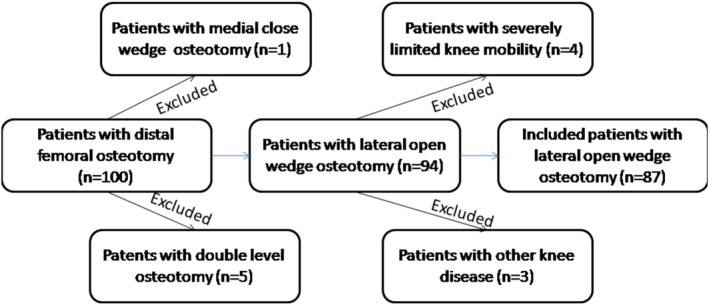
Flow chart for patients inclusion and exclusion in this research.

This research was approved by the local institutional review board, and informed written consents were given to all the patients (protocal number 2021‐014). We retrospectively studied a total of 100 patients who were admitted to our hospital for the treatment of genu valgum using LOWDFO. To improve the accuracy of the surgery, we used a 3D printed patient specific instrument (PSI) designed based on a preoperative computed tomography (CT) scan (Figure 1). This made the surgical procedure more precise and excluded the influence of factors such as surgical operation proficiency. Another aim of using PSI in this study is to avoid excessive gap opening and osteotomy depth during the surgery, which may pose a higher risk of hinge fracture and influence the results of this study.

### 2.1 Inclusion Criteria and Exclusion Criteria for Patients

The patients admitted to our hospital from January 1st,2022 to January 1st,2024 with genu valgum deformity and lateral unicompartmental osteoarthritis treated with distal femoral osteotomy were in this study. The minimum follow‐up period for inclusion criteria was 6 months. The inclusion criteria: (i) patients with genu valgum deformity and lateral unicompartmental osteoarthritis treated with LOWDFO. (ii) These patients' MAD were below −10 mm. (iii) mLDFA were below 85°. The exclusion criteria: (i) patients with inflammatory arthropathies. (ii) Patients with multicompartmental arthritis. (iii) Patients with severe limitation of knee flexion and extension (preoperative knee mobility < 100°, flexion contracture > 15°). (iv) Patients who underwent LOWDFO along with other orthopedic surgeries such as tibial medial close wedge osteotomy were excluded (Figure 1).

### 2.2 Design and Production of PSI

The surgery was simulated on a computer based on the patient's preoperative CT data. The distal lateral femoral planned opening angle and the depth of the osteotomy gap were calculated based on the reversed Miniaci's method [12]. The designed digital model of orthopedic positioning slice and filler block in STL format was imported into the 3D printer EOS P110. The printing process was selective laser sintering (SLS), and the printing material was imported into the 3D printing slicing and layering software PSW FORMIGA. The 3D printing parameters were designed, and the printing accuracy was 0.625 mm. The 110 Velocis laser sintering printing instrument used the printing material PA 2000 nylon, and the printing accuracy was 0.625 mm.

### 2.3 Surgical Procedure

**FIGURE 2:**
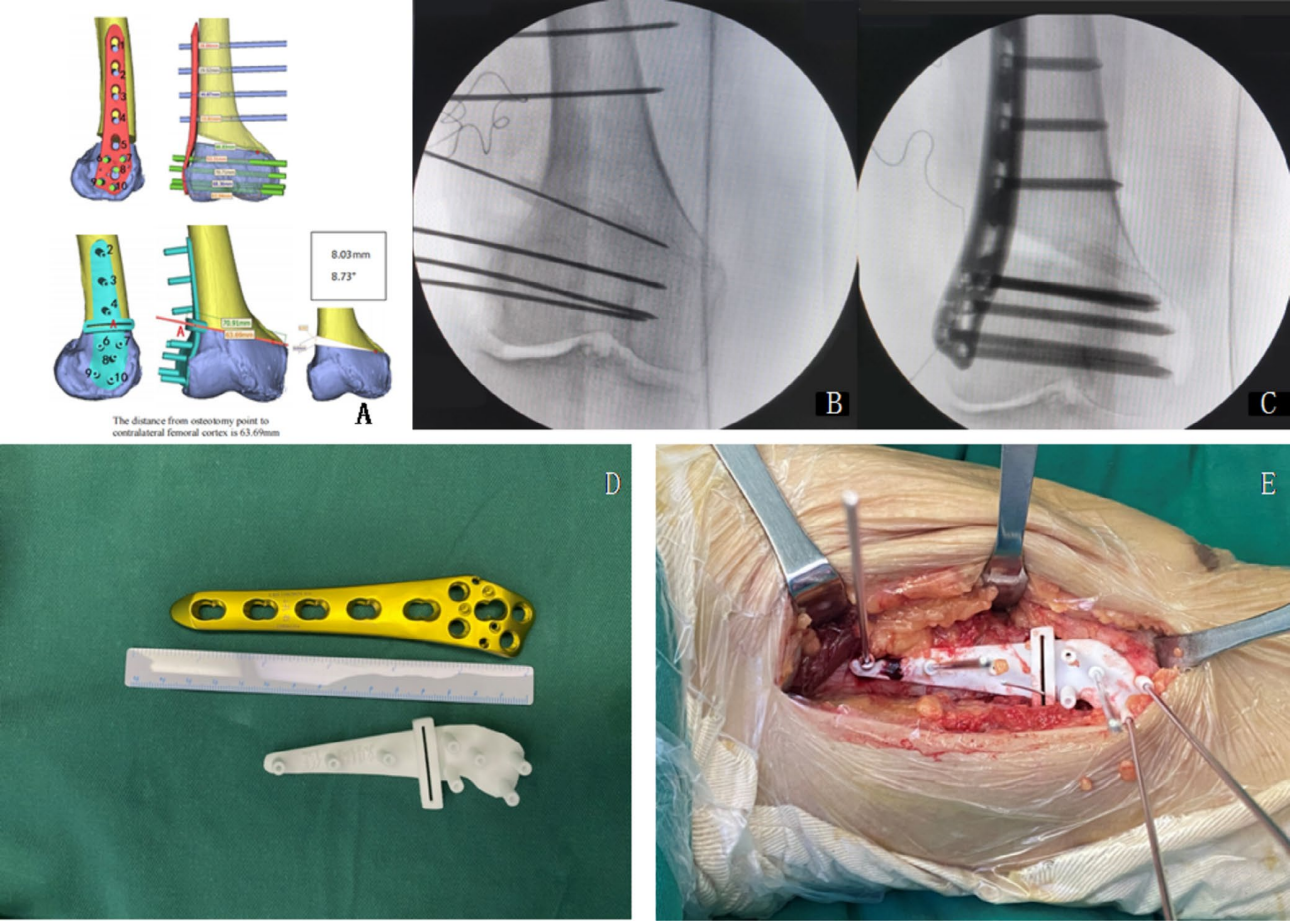
(A) 3D osteotomy planning based on preoperative CT scan. (B) Intraoperative fluoroscopic images. Adjustment to the optimal osteotomy position according to intraoperative fluoroscopic images. (C) Intraoperative fluoroscopic images. The bone is cut, and the gap is opened, plate screws are placed, and intraoperative fluoroscopy is performed to ensure surgical precision. (D) Titanium plate and 3D patient‐specific printed cutting guide used in the surgery procedure. (E) Intraoperative use of 3D patient‐specific printed cutting guide to determine the direction of osteotomy.

**FIGURE 3:**
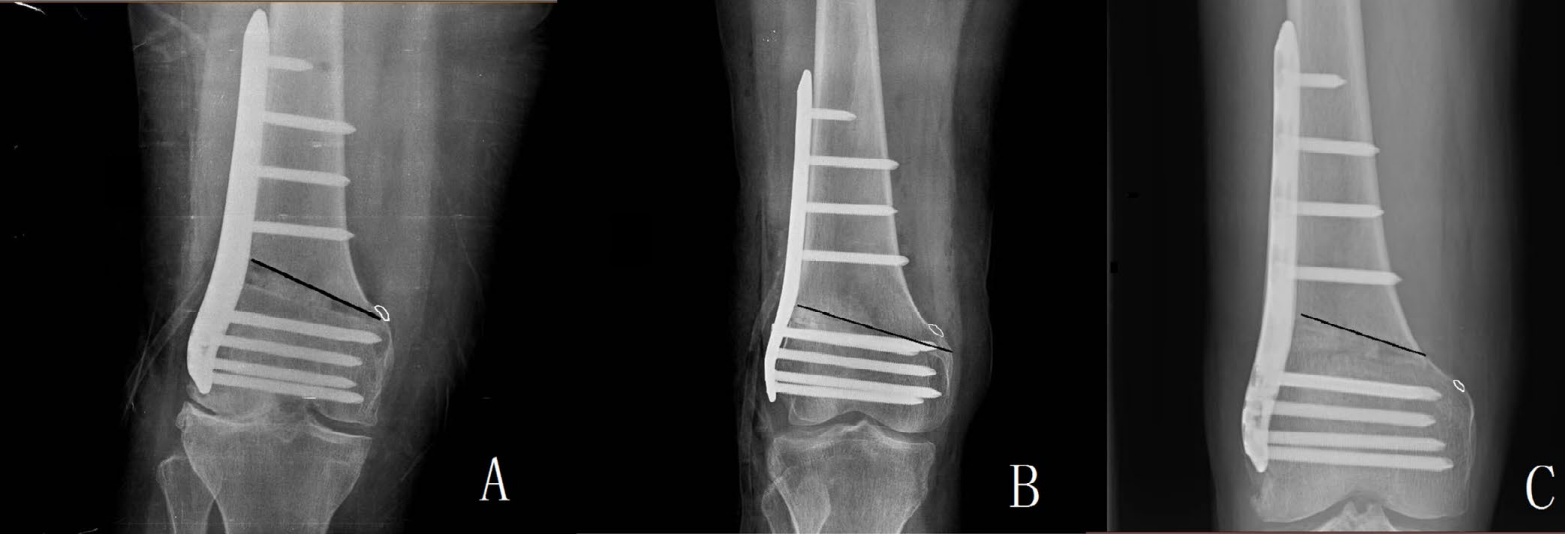
Relationship between the osteotomy line and the adductor tubercle. The white circle marks the position of the adductor tubercle and the black line marks the position of the osteotomy line. The intersection of the osteotomy line and the cortex is the hinge point. Group A: hinge point localized at the adductor tubercle. Group B: hinge point localized proximal to the adductor tubercle. Group C: hinge point localized distal to the adductor tubercle.

**FIGURE 4:**
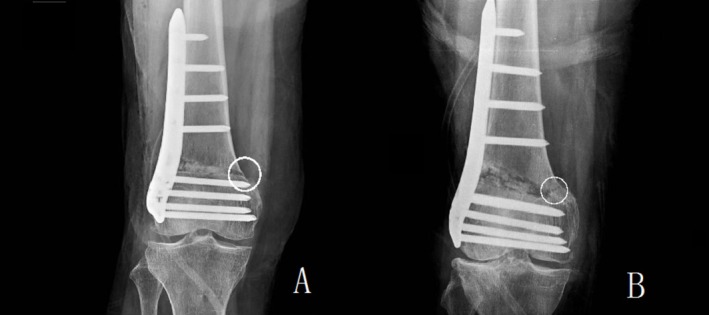
Type 1 fracture means the fracture line is an extension of the osteotomy line that reaches the hinge. Type 2 fracture means the fracture line extends toward the distal femur. Type 3 fracture means the fracture line extends toward the proximal femur. To our data, there are only 2 types of hinge fracture: Group A: Type 1 fracture. Group B: Type 3 fracture.

The corresponding author was the lead surgeon. The first and the second authors performed the surgery as assistants. With the patient in the supine position, general anesthesia was administered by an anesthetist. The surgeon made the distal lateral straight incision. The incision length was about 14 cm. We cut through the skin, subcutaneous tissue, iliotibial fascia, and made an incision at the posterior lateral interspace of the lateral femoral muscle to expose the distal femur. We placed the 3D printed patient specific instrument (PSI) for cutting guide (Figure 2). We used five K‐wires (*φ* = 2.0 mm) to fix the cutting guide to the femur and used one K‐wire (*φ* = 1.2 mm) to mark the direction of the osteotomy (Figures 3 and 4). We used intraoperative fluoroscopy to determine that the direction of the osteotomy was aimed toward the AT. The surgeon made the osteotomy according to the direction of the cutting guide. He took out the cutting guide. According to the directions of the five K‐wires, we placed the titanium plate (Ruihe distal femoral locking plate, China) and inserted the sockets and the cores of the sockets into the hole of the titanium plate. Then the gap was spread to the preoperatively planned distance using a spreader. We fixed the titanium plate with screws of the right length. We performed arthroscopy to clean the joint cavity of diseased tissue. The surgeon performed meniscoplasty and synovial cleaning if necessary to reduce the risk of postoperative pain. We performed allogeneic bone grafting (bio‐gene, Datsing, China). According to postoperative radiological data, the line of force of the lower limb adjusted to the center of the joint meant the surgery was successful [13, 14, 15]. The patients began rehabilitation in bed after recovery from anesthesia. Partial weight‐bearing training was started at 4 weeks postoperatively, and normal floor training was started at 6 weeks postoperatively.

### 2.4 Clinical Parameters and Radiological Data Collection

Anteroposterior long‐leg radiographs of both lower limbs in the standing position, lateral films of the affected knee, CT scans of both lower limbs and magnetic resonance imaging (MRI) of the affected knee were taken in all patients before surgery [16]. Multiple parameters were included in this study. The first author and the second author observed and recorded the data separately. If observations were inconsistent, other authors discussed together to decide on reasonable values. We use Intraclass correlation (ICC) method to test the consistency of the radiological data measured by the first two authors. We used the ICC method to test the consistency of the actual opening distance (mm), the actual opening degree (°), the Insall salvati index and the post‐op (post operative) lower limb lengthening (mm) and found these four parameters were in good consistency (the single measures of ICC were 0.998, 1.000, 0.999, and 0.995), if the ICC > 0.700, the data should be considered in good consistency. Basic clinical parameters available preoperatively including age at time of surgery, sex, laterality, height, body weight, body mass index (BMI), Insall Salvati Index, preoperative and postoperative WOMAC (The Western Ontario and McMaster Universities Osteoarthritis Index, preoperative WOMAC was collected just before surgery, postoperative WOMAC was collected 6 months after surgery), and postoperative lower limb lengthening. We collected the preoperative and postoperative Insall Salvati index of the patients by lateral X‐ray. Complications such as delayed union, infection, symptoms of iliotibial band irritation, deep vein thrombosis and comorbidities, such as osteoporosis, Type 2 diabetes mellitus, and post total thyroidectomy which may affect postoperative outcomes, were comprehensively collected and listed in Table 1. For the notation of continuous data, if the data are normally distributed after testing for normality, we used “mean ± SD”, for data that was not normally distributed, we used “median (interquartile range)”. Data related to osteotomies were presented in Table 2. Based on the location of the hinge point, the study patients were divided into 3 groups. Group A: hinge point localized at the adductor tubercle. Group B: hinge point localized proximal to the adductor tubercle. Group C: hinge point localized distal to the adductor tubercle (Figure 3). Thanks to the 3D osteotomy cutting guide technology, we can measure the planned opening distance, the actual opening distance, the planned opening degree, and the actual opening degree. MAD is the perpendicular distance between the mechanical axis of the lower limb and the centre point of the knee joint. The mechanical axis deviation pre‐operation was recorded as pre‐op MAD. The mechanical axis deviation post‐operation was recorded as post‐op MAD. MAD correction means post‐op MAD minus pre‐op MAD. MAD correction/丨pre‐op MAD丨was defined as MAD correction ratio. We uniquely defined the ratio of MAD corrected value/absolute pre‐operative MAD value as MAD correction ratio. Since some patients corrected through the midpoint of the joint, we recorded the valgus MAD value as negative and the varus MAD value as positive for ease of arithmetic. We recorded preoperative mLDFA values and postoperative mLDFA values and derived the mLDFA correction values by subtracting the two. Mechanical lateral distal femur angle was recorded as mLDFA. Similar to MAD, we recorded pre‐op mLDFA, post‐op mLDFA, mLDFA correction and mLDFA correction ratio. mLDFA correction/pre‐op mLDFA was defined as mLDFA correction ratio. Parameters related to fractures were listed in Table 3. Some researchers have proposed a number of ways of typing hinge fractures [8, 9]. Type 1 fracture means the fracture line is an extension of the osteotomy line that reaches the hinge. Type 2 fracture means the fracture line extends toward the distal femur. Type 3 fracture means the fracture line extends toward the proximal femur (Figure 4). According to our data, we found no type 2 fractures. We took plain films of the knee at 2 weeks postoperatively and full‐length images of the lower limb at 6 weeks postoperatively. Healing time was defined as the moment when the osteotomy line and the fracture line blur on full‐length images of the lower limb. If there were hinge fractures (IHF or PHF) we will review full‐length films of the lower extremity every 4 weeks. A clear osteotomy line or fracture line still visible on X‐ray 3 months after surgery was defined as delayed healing. If the healing time exceeded 9 months, it was defined as nonunion.

**TABLE 1:** Basic clinical data of the total study group.

Variable	Total study group
Number of included patients, *n*	87
Age (years)	58.64 ± 9.61 (27–77)
Sex	
Male, *n* (%)	11 (12.64%)
Female, *n* (%)	76 (87.36%)
Laterality	
Right, *n* (%)	60 (68.97%)
Left, *n* (%)	18 (31.03%)
BMI (kg/m^2^)	23.03 ± 1.66 (19.49–28.67)
Insall salvati index	1.179 ± 0.236 (0.600–1.795)
Pre‐op WOMAC	26.460 ± 13.837 (3–78)
Post‐op WOMAC	9.700 ± 8.181 (0–50)
Post‐op lower limb lengthening (mm)	6.379 ± 7.917 (0–41)
Complications *n* (%)	
Delayed‐union	36 (41.38%)
Infection	1 (1.15%)
Symptoms of iliotibial band irritation	87 (100%)
Deep vein thrombosis	25 (28.74%)
Comorbiditis *n* (%)	
Osteoporosis	25 (28.74%)
Type 2 diabetes mellitus	9 (10.34%)
Total thyroidectomy	4 (4.60%)

**TABLE 2:** Parameters related to osteotomy.

Variable	Total study group
Number of included patients *n*	87
Hinge position	
Proximal to the adductor tubercle *n* (%)	10 (11.49%)
Adductor tubercle *n* (%)	65 (74.72%)
Distal to the adductor tubercle *n* (%)	12 (13.79%)
Planned opening distance (mm)	9.769 ± 4.396 (4.07–23.59)
Actual opening distance (mm)	11.907 ± 3.893 (4.56–27.00)
Planned opening degree (°)	9.575 ± 4.290 (4.22–22.00)
Actual opening degree (°)	11.861 ± 3.893 (4.43–24.89)
Pre‐op MAD (mm)	−30.544 ± 17.975^a^ (−113.00 to −11.00)
Post‐op MAD (mm)	−1.277 ± 9.757 (−46.00 to 14.31)
MAD correction (mm)	29.267 ± 17.388 (7.40–111.90)
MAD correction ratio^b^	0.969 ± 0.375 (0.36–2.00)
Pre‐op mLDFA (°)	83.308 ± 3.005 (70.67–89.78)
Post‐op mLDFA (°)	91.876 ± 3.525 (81.00–100.00)
mLDFA correction (°)	8.695 ± 3.665 (1.28–22.18)
mLDFA correction ratio^c^	0.105 ± 0.047 (0.02–0.29)

^a^ The MAD of the valgus knee was recorded as a negative number, MAD of the varus knee was recorded as a positive number. The absolute value representing the severity of valgus or varus. This method makes it easier to calculate the corrected value.

^b^ MAD correction/丨pre‐op MAD丨is defined as MAD correction ratio.

^c^ mLDFA correction/pre‐op mLDFA is defined as mLDFA correction ratio.

**TABLE 3:** Parameters related to fractures.

Variable	Total study group
Number of included patients *n*	87
Total number of patients with intraoperative and postoperative fractures	36 (41.379%)
Intraoperative fractures	20 (55.556%)
Fracture morphology	
Type 1 (extension), *n* (%)	8 (40.000%)
Type 2 (distal), *n* (%)	0 (0.000%)
Type 3 (proximal), *n* (%)	12 (60.000%)
Postoperative fractures	16 (44.444%)
Fracture morphology	
Type 1 (extension), *n* (%)	12 (75.000%)
Type 2 (distal), *n* (%)	0 (0.000%)
Type 3 (proximal), *n* (%)	4 (25.000%)
Healing time (months)	3.052 ± 0.986
Delayed union *n* (%)^a^	36 (41.379%)

^a^ The patient is considered to have delayed union if the healing time is more than 3 months.

## 3 Statistical Analysis

Statistical analysis was conducted using SPSS software version 19 (IBM, New York, USA) with a significance level set at *p* < 0.05. Sample size estimation was performed using the formula: $n=\frac{{\left(Z_{\alpha }+Z_{\beta }\right)}^{2}\times 2\sigma^{2}}{\delta^{2}}$ where *n* is the sample size per group, power = 0.8, *α* = 0.05, Z*α* + Z*β* = 1.96 + 1, *σ* is the standard deviation, and *δ* is the difference between group means. Further analyses were performed only when the sample size met or exceeded this estimate. Continuous variables were expressed as mean ± standard deviation (SD) if normally distributed, or as median (interquartile range) if not. Normality was assessed by the *F*‐test, with *p* ≥ 0.05 indicating normal distribution. Independent samples *t*‐tests compared continuous variables between groups, while paired‐sample *t*‐tests assessed preoperative and postoperative changes. Categorical variables were presented as counts and percentages; comparisons used the Chi‐square test, or Fisher's exact test if expected cell frequencies were less than 1. Since the direction of the osteotomy is from outside to inside, the direction of the osteotomy line is not as precise as the medial side, we defined the osteotomy line pointing to AT, 5 cm proximal to AT, and 5 cm distal to AT as categorical variables and analyzed these as categorical variables using Chi‐square tests. We compared all of the individual categorical variables in correlation. As the vast majority of the data were not correlated with PHF, we did not need to perform multiple regression analyses. All comparisons of measures were first subjected to normality testing, and we used the *F*‐test, where a *p*‐value greater than or equal to 0.05 was considered normally distributed. For data affected by multiple factors, collinearity diagnosis was first performed. If the variance inflation factor (VIF) was larger than 10, eliminate the collinear variables [17]. A binary logistic regression was performed to determine the odds and 95% confidence interval.

## 4 Results

### 4.1 Basic Clinical Data of the Total Study Group

We collected 100 patients' data with distal femoral osteotomy. A total of 87 patients with genu valgum requiring LOWDFO were included in this study. The follow‐up period was 6 months, and the follow‐up rate was 100%. We used the mean of these parameters to do further comparisons. The basic clinical data of these patients was shown in Table 1. The Insall Salvati index was 1.18 ± 0.24, and we found no difference between groups. In Table 4, we found that there was a statistically significant difference between the pre‐operation data and the post‐operation data, and the patient's postoperative WOMAC score was significantly reduced, *p* < 0.001. We studied the patient's postoperative affected limb lengthening (6.4 ± 7.9 mm), but did not find any association with the affected limb lengthening. For complications, we mainly collected data regarding delayed healing, infection, iliotibial band irritation, and deep vein thrombosis. Patients developed lower extremity deep vein thrombosis postoperatively, but the incidence of thrombosis was not statistically related to any of the clinical indicators we studied. We studied three comorbidities: osteoporosis, type 2 diabetes mellitus, and post‐total thyroidectomy. Using the Chi square method, we did not find any association of these factors with IHF or PHF by comparison between groups.

**TABLE 4:** Paired *t*‐tests comparing pre‐operation and post‐operation variables.

Variable	Pre‐operation	Post‐operation	*p*
MAD (mm)	−30.544 ± 17.975	−1.898 ± 10.940	< 0.001
mLDFA (degree)	83.308 ± 3.005	91.876 ± 3.525	< 0.001
WOMAC	26.460 ± 13.837	9.700 ± 8.181	< 0.001

*Note*: The MAD, mLDFA, and WOMAC are compared to verify satisfactory surgical results.

### 4.2 Parameters Related to Osteotomy

It showed that with the help of 3D osteotomy cutting guides, precise osteotomies can be achieved in most patients. We collected preoperative MAD values. Postoperative MAD values were shown in Table 4, where we performed a paired samples t‐test and found MAD was significantly reduced. The MAD values were corrected from −30.5 ± 18.0 mm preoperatively to −1.3 ± 9.8 mm postoperatively (*p* < 0.001). The corrected MAD was obtained by subtracting the two values. Table 3 depicted the parameters associated with IHF and PHF. All the hinge fractures were 36, which was 41.4% of all the patients. There were 20 patients with intraoperative fractures and 16 patients with postoperative fractures. To our data, 36 patients experienced delayed union, but no patient had bone nonunion. Due to the large number of statistics, we have listed only statistically significant items in Table 5. Hinge fractures significantly affected healing time and correlated with Post‐op mLDFA, MAD correction, mLDFA correction, and body weight.

**TABLE 5:** Group comparison (hinge fractures verusus no hinge fractures).

Variable	Hinge fracture		*p*
	Yes	No	
Number of patients *n*	36	51	
Healing time (months)	3.361 ± 1.223	2.833 ± 0.712	0.013
Post‐op mLDFA (°)	93.220 ± 3.342	91.143 ± 3.107	0.004
MAD correction (mm)	34.940 ± 20.367	25.262 ± 13.788	0.010
mLDFA correction (°)	10.144 ± 3.863	7.672 ± 3.174	0.002
mLDFA correction ratio	0.123 ± 0.050	0.093 ± 0.040	0.002
Body weight (kg)	64.417 ± 7.489	60.549 ± 6.338	0.011

*Note*: Patients with hinge fractures including intraoperative and postoperative fractures are compared with no hinge fractures patients, only statistically significant variables are represented in the table. We use these data for binary logistic regression in the Supporting Information S1.

### 4.3 Parameters Related to Hinge Fractures

We compared the IHF group with the no IHF group, and statistically significant data was recorded in Table 6. The healing time of patients in the IHF group (3.9 ± 1.4 months) was longer than that in the no intraoperative hinge fractures group (2.8 ± 0.7 months, *p* < 0.001). The mLDFA correction in the IHF group (10.7° ± 4.3°) was significantly higher than the mLDFA in the no IHF group (8.1° ± 3.4°, *p* = 0.005). The mLDFA correction ratio in the IHF group (0.131 ± 0.056) was significantly higher than that in the no IHF group (0.098 ± 0.041, *p* = 0.005). In addition, the height, body weight, and BMI of the IHF group were significantly higher than those of the no intraoperative fractures group, with *p*‐values of 0.032, 0.002, and 0.031, respectively. There was a significant difference between the sexes. Using the chi‐square test for counting data, the proportion of males in the IHF group was significantly higher than that of the no IHF group, with *p* = 0.008. Male patients were more likely to have IHF. Using these correlations, we performed binary logistic regression. Regression equation: y(IHF) = 0.247*mLDFA correction −1.236*sex + 0.440*BMI −11.465 (The data collinearity diagnosis and binary logistic regression process were documented in Supporting Information S2). Intraoperative hinge fracture prolonged the healing time and correlates with mLDFA correction, sex, and BMI.

**TABLE 6:** Group comparison (intraoperative hinge fracture versus no intraoperative hinge fracture).

Variable	Intraoperative hinge fractures		*p*
	Yes	No	
Number of patients *n*	20	67	
Healing time (months)	3.900 ± 1.392	2.799 ± 0.652	< 0.001
mLDFA correction (°)	10.676 ± 4.322	8.104 ± 3.253	0.005
mLDFA correction ratio	0.131 ± 0.056	0.098 ± 0.041	0.005
Height (m)	1.671 ± 0.077	1.632 ± 0.067	0.032
Body weight (kg)	66.450 ± 8.172	60.866 ± 6.201	0.002
BMI	23.729 ± 1.721	22.821 ± 1.597	0.031
Sex			
Male *n*	6	5	0.008
Female *n*	14	62	

*Note*: Only statistically significant variables are represented in the table. We use these data for binary logistic regression in the Supporting Information S2. Regression equation: y(IHF) = 0.247*mLDFA correction −1.236*sex + 0.440*BMI −11.465.

We compared the PHF group with the no PHF group and found hinge position was associated with postoperative hinge fractures (Table 7). A 3 × 2 chi‐square test was performed; PHF was related to hinge position (*p* = 0.001). Then we used a 2 × 2 chi‐square test to analyze the data separately. The PHF risk of the hinge localized proximal to the adductor tubercle was analyzed using the hinge in the adductor tubercle as a reference, *p* < 0.001 OR = 10.688, and the 95% confidence interval was 2.468–46.282. The PHF risk of the hinge localized distal to the adductor tubercle was compared to the hinge localized to the adductor tubercle group using a 2 × 2 chi‐square test (*p* = 0.680). OR = 1.425, and the 95% confidence interval is 0.262–7.714. Thus, the hinge localized proximal to the adductor tubercle was associated with postoperative fracture, whereas there was no statistically significant difference in postoperative fracture when the hinge localized distal to the adductor tubercle.

**TABLE 7:** Group comparison (postoperative hinge fracture versus no postoperative hinge fracture).

Variable	Hinge fracture		*p*
	Yes	No	
Number of patients *n*	16	71	
Hinge position			
Proximal to the adductor tubercle *n*	6	4	0.001
Adductor tubercle *n*	8	57	
Distal to the adductor tubercle *n*	2	10	

IHF occurred before PHF. To avoid IHF data influencing the PHF study, we excluded IHF patients and compared the PHF group with the no hinge fracture group (Table 8). The hinge position was associated with postoperative hinge fracture. A 3 × 2 chi‐square test was performed; postoperative hinge fractures were related with hinge position (*p* = 0.001). Then we used a 2 × 2 chi‐square test to analyze the data separately. The postoperative hinge fractures of the hinge proximal to the adductor tubercle was analyzed using the hinge in the adductor tubercle as a reference, *p* < 0.001, OR = 15.375, and the 95% confidence interval is 2.617 ~ 90.314. The hinge localized to distal to the adductor tubercle group was compared to the hinge localized to the adductor tubercle group using a 2 × 2 chi‐square test (*p* = 0.778). OR = 1.281 and the 95% confidence interval was 0.228–7.189. The hinge localized proximal to the adductor tubercle was associated with postoperative fractures, whereas there was no statistically significant difference in postoperative fractures when the hinge localized distal to the adductor tubercle. Our data was consistent with the findings of Winkler's research [11]. In addition, we found that postoperative hinge fractures were associated with postoperative mLDFA. The mLDFA was larger in the PHF group (93.5° ± 3.2°) than the mLDFA with the no hinge fracture group (91.1° ± 3.1°, *p* = 0.011). PHF did not affect healing time (*p* = 0.493).

**TABLE 8:** Group comparison (postoperative hinge fracture versus no hinge fracture).

Variable	Hinge fractures		*p*
	PHF no hinge fracture		
Number of patients *n*	16	51	
Healing time (months)	2.688 ± 0.403	2.833 ± 0.712	0.439
Postoperative mLDFA (°)	93.472 ± 3.151	91.143 ± 3.107	0.011
Hinge position			
Proximal to the adductor tubercle *n*	6	2	0.001
Adductor tubercle *n*	8	41	
Distal to the adductor tubercle *n*	2	8	

We performed an independent samples t‐test between the data of IHF group and PHF group (Table 9). It was found that the healing time in the IHF group (3.9 ± 1.4 months) was significantly longer than the healing time in the postoperative fracture group (2.7 ± 0.4 months. *p* = 0.002). There were no statistical differences between types of fractures in terms of their impact on healing time, regardless of whether the fractures were IHF or PHF.

**TABLE 9:** Group comparison (intraoperative hinge fractures versus postoperative hinge fractures).

Variable	Hinge fractures		*p*
	Intraoperative postoperative		
Number of patients *n*	20	16	
Healing time (months)	3.900 ± 1.392	2.688 ± 0.403	0.002

*Note*: Patients with intraoperative hinge fractures (3.900 ± 1.392) take longer time to heal than those with postoperative hinge fractures (2.688 ± 0.403). *p* = 0.002.

## 5 Discussion

### 5.1 Main Finding

The main finding of our study was that during LOWDFO treatment of genu valgum, IHF and PHF had different causes. IHF was primarily associated with corrected angulation during the surgical procedure, and body weight was related. PHF was mainly related to hinge position. For the first time, we examined the causes and outcomes of IHF and PHF in LOWDFO separately. More precise conclusions were obtained. These two types of fractures should be distinguished for future studies to be more convincing.

### 5.2 Are Factors Affecting IHF and PHF the Same?

If we consider IHF and PHF in LOWDFO as one event, clinical parameters and radiological data should be analyzed together. Postoperative mLDFA in the hinge fracture group was larger than in the no hinge fracture group. This result suggested that overcorrection increased the risk of hinge fractures. We recommend that postoperative mLDFA should be less than 93° to reduce the risk of hinge fractures. MAD correction, mLDFA correction, and mLDFA correction ratio are related to patient selection. We recommend that if the MAD correction is more than 35 mm, the mLDFA correction is more than 10°or the mLDFA correction ratio is more than 0.123 (12.3%), According to Paley's principles for deformity correction. The center of rotation angulation (CORA) that determines the surgical approaches should be determined according to the malalignment of the limb. Misuse of this osteotomy will lead to an oblique joint line [16]. Such patients should be informed of the increased likelihood of fractures before surgery. Maybe double‐level osteotomies and early body weight control should be considered.

If we consider IHF and PHF as different events in LOWDFO, they should be analyzed separately. It is easily understood that intraoperative hinge fractures prolonged the healing time. The mLDFA correction greater than 10.7°or the mLDFA correction ratio greater than 0.131 (13.1%) may lead to intraoperative hinge fractures. The IHF was related to BMI; body weight control should be recommended before surgery. Furthermore, we found that gender was related to intraoperative hinge fractures. Male patients had a high risk of IHF. There was no literature to support this result. We will delve into this interesting phenomenon in future studies.

After analyzing the parameters regarding PHF, we found that most of them were irrelevant, even though they were found significant in the research on IHF. The hinge position was found to be relevant. It was therefore reasonable to assume that IHF and PHF were two different clinical events. Based on our data, the hinge localized proximal to the adductor tubercle was associated with postoperative fractures. The cortex in this place is thickened but fragile. To avoid postoperative fractures, patients whose hinge localized proximal to the adductor tubercle should be recommended to lie in bed for more time. According to our data, patients with IHF (3.9 ± 1.4 months) took longer to heal than those with PHF (2.7 ± 0.4 months, *p* = 0.002). Both IHF and PHF should be avoided, while delayed bone healing should be paid special attention for IHF. In fact, for open wedge corrective osteotomies, there are many methods that may help to enhance the stability of the bone‐implant construct and avoid hinge fracture. For example, additional screw passing across the opening gap. The start of loading should be delayed. However, the range of motion training should not be delayed.

### 5.3 Other Findings

There were no type 2 hinge fractures in our collected sample. According to our data, there was no statistical difference in all aspects of patient's postoperative recovery, healing time, etc., when comparing different types of hinge fractures. Hinge fracture type may influence patient recovery after HTO; however, we did not find similar results. Some research about hinge fractures in lateral closed‐wedge distal femoral osteotomy in knees inspires us [18]. Morphology of hinge fractures related to LOWDFO was not that important. Madelaine et al. reported that LOWDFO had no effect on leg length [19]. However, to our data, after LOWDFO, the affected leg is prolonged 6.4 ± 8.0 mm. If the lower limb does not lengthen by more than 2 cm, this gap can be compensated for by pelvic tilt; therefore, while LOWDFO lengthens the affected limb, the amount of lengthening does not exceed what the body can tolerate. We record the full comparative data in Supporting Information S3, including both positive and negative results, as the important negative data results are also relevant to many readers.

Neither IHF nor PHF affected the WOMAC score at 6 months' time. Thus, IHF and PHF only affect the speed of recovery, but not the surgical outcomes.

### 5.4 Prospects for LOWDFO Applications

Why did knee valgus occur? Many researchers believed that genu valgum was associated with lateral meniscus injury and lateral meniscectomy. The procedure of lateral meniscectomy was linked to an increased likelihood and speed of degeneration when compared with medial meniscectomy [14, 20, 21]. However, Liles found medial closing wedge high tibial osteotomy (MCWHTO) was able to unload the lateral compartment more effectively compared with LOWDFO [22]. Femoral deformity and tibial deformity have different causes; we advise that MCWHTO is not a substitute for LOWDFO. LOWDFO lengthens the length of the affected limb, whereas MCWDFO shortens the length of the affected limb. If the affected limb is shorter than the contralateral side, we have to choose LOWDFO, and therefore this procedure has an important medical value.

We found postoperative iliotibial band irritation signs occurred in all patients, which were higher than the results found by Saithna et al. [23]. Jacobi et al. reported local irritation of the iliotibial tract requiring implant removal in 86% of the patients with the Tomofix plate [1]. For these reasons, few surgeons prefer to use the LOWDFO approach to treat genu valgum. LOWDFO stands out as a precise and effective method for rectifying symptomatic deformities in genu valgum. Additionally, surgery has led to a notable enhancement in patients' outcomes [6]. Utilizing LOWDFO with locking plate fixation proves to be a dependable and precise method in treating symptomatic genu valgum. The method offers enhanced pain relief and better functional results for patients, along with consistent bone repair at the site of osteotomy [17]. LOWDFO was once rarely used by surgeons due to complications and other reasons. However, based on in‐depth studies by more scientists, it has been found that this procedure has many advantages and brings more benefits to patients than the risk of complications. The advantage of LOWDFO over medial closure osteotomy of the distal femur is that after performing the osteotomy, it is still possible to adjust the spreading gap, which improves surgical tolerance and avoids inadequate corrections or overcorrections. It is worthwhile to conduct in‐depth research to reduce the risk of complications for the benefit of patients.

### 5.5 Strengths and Limitations

There are some strengths of the study design. Separate studies of intraoperative and postoperative fractures can avoid interference between these two types of fractures. The use of digital orthopedic technology to improve surgical precision and reduce the impact of surgical operative factors on results is beneficial.

There are some limitations to this study. In this study, selection bias was generated due to an improper sample selection method as random sampling was not used. We will conduct a randomized controlled experiment to reduce selection bias in future research. Two authors measured the data separately in order to reduce measurement bias, and then calculated the ICC, and if the measurements differed significantly, we asked a third author to participate in the measurement. Confounding factors such as age and gender may influence conclusions. We stratified the analysis by adjusting for possible confounding factors; the ORs before and after correction in our data did not differ significantly, and it can be assumed that these are not confounders.

LOWDFO is a treatment for valgus knees, but due to the need for bone grafts and the very high level of iliotibial bundle irritation symptoms, this method is not well accepted and reduces the generalizability. Our study included 87 samples. Eighty seven meets the requirements for sample size estimation, but the sample could continue to be collected to improve confidence. Our study was a single‐centre study, and the sample collected was patients admitted to our hospital. In order for the study to have a higher efficacy, a multi‐centre study can be achieved in future studies through collaboration of multiple hospitals. This study was a retrospective study with a lower grade of evidence than prospective studies. The follow‐up period in this study was 6 months, and the short follow‐up period reduces the reliability of the data.

## 6 Conclusion

IHF correlates with mLDFA correction, sex, and BMI and prolongs osteotomy healing time. Unlike IHF, PHF correlates with hinge position. The hinge position localized to the proximal of the AT increases the incidence of PHF; however, PHF does not affect healing time. Preoperatively planned high mLDFA correction and high mLDFA correction ratio are related to IHF. These kinds of patients should be informed of the increased likelihood of delayed union. Preoperative body weight control is recommended for patients requiring LOWDFO to reduce IHF incidence and shorten healing time. Patients with hinge points localized proximal to the AT should be informed of the increased incidence of PHF, and early weight bearing on the floor should be avoided. For LOWDFO, there is no statistical difference between fracture types (Type 1 or Type 3) in terms of their impact on healing time, regardless of whether the fracture is an IHF or a PHF. IHF and PHF are different in terms of associated factors and outcomes.

## Author Contributions

**Zijian Lian:** conceptualization, data curation, methodology, writing – original draft. **Bin Zhao:** conceptualization, investigation, methodology, writing – review and editing. **Jianxiong Ma:** data curation. **Songqing Ye:** data curation, funding acquisition. **Haohao Bai:** funding acquisition, validation. **Zhihu Zhao:** validation. **Xuan Jiang:** writing – review and editing. **Fei Xing:** writing – review and editing. **Yao Deng:** writing – review and editing. **Wei Luo:** funding acquisition, validation, writing – review and editing. **Xinlong Ma:** funding acquisition, validation, writing – review and editing.

## Conflicts of Interest

The authors declare no conflicts of interest.

## Supporting information


**Data S1:** Supporting Information.

**Data S2:** Supporting Information.

**Data S3:** Supporting Information.
