# Supplementary material for: Analysis of Intraoperative and Postoperative Hinge Fractures of Patients With Genu Valgum Treated With Lateral Open Wedge Distal Femoral Osteotomy
Source: Orthop Surg. 2025 Aug 5;17(9):2629–39. doi: 10.1111/os.70142 (PMC12404878; doi:10.1111/os.70142)
Supplement: Supplementary file 1 — Data S1: Supporting Information. [file OS-17-2629-s003.docx]

|  | | B | S.E. | t | sig | tolerance | VIF |
| --- | --- | --- | --- | --- | --- | --- | --- |
|  | (constant) | -1.770 | 3.864 | -0.458 | 0.648 |  |  |
|  | Post-op mLDFA | 0.004 | 0.046 | 0.097 | 0.923 | 0.100 | 9.953 |
|  | MAD correction | 0.002 | 0.005 | 0.389 | 0.699 | 0.374 | 2.671 |
|  | mLDFA correction | 0.165 | 0.408 | 0.405 | 0.686 | 0.001 | 947.906 |
|  | mLDFA correction ratio | -9.755 | 30.395 | -0.321 | 0.749 | 0.001 | 854.518 |
|  | Body weight | 0.021 | 0.007 | 2.960 | 0.004 | 0.932 | 1.073 |

Collinearity statistics show the mLDFA correction, mLDFA correction ratio’ VIF are more than 10 which means these independent variables have collinearity. We eliminated mLDFA correction ratio. We use Post-op mLDFA, MAD correction, mLDFA correction and body weight to do collinearity statistics again.

|  | | B | S.E. | t | sig | tolerance | VIF |
| --- | --- | --- | --- | --- | --- | --- | --- |
|  | (constant) | -2.878 | 1.724 | -1.670 | .099 |  |  |
|  | Post-op mLDFA | .018 | .020 | .878 | .383 | .516 | 1.938 |
|  | MAD correction | .002 | .004 | .343 | .732 | .384 | 2.602 |
|  | mLDFA correction | .035 | .027 | 1.305 | .196 | .245 | 4.086 |
|  | Body weight | .021 | .007 | 3.013 | .003 | .939 | 1.065 |

This time, all VIF are smaller than 10.We use Post-op mLDFA, MAD correction, mLDFA correction and Body weight to do binary logistic regression comparing hinge fractures group vs. no hinge fractures group.

|  | | B | S.E, | Wals | Sig. | Exp (B) | EXP(B) 95% C.I. | |
| --- | --- | --- | --- | --- | --- | --- | --- | --- |
|  |  |  |  |  |  |  | lower | upper |
|  | Post-op mLDFA | 0.102 | 0.106 | 0.927 | 0.336 | 1.107 | 0.900 | 1.363 |
|  | MAD correction | 0.017 | 0.027 | 0.425 | 0.514 | 1.018 | 0.966 | 1.072 |
|  | mLDFA correction | 0.161 | 0.147 | 1.202 | 0.273 | 1.174 | 0.881 | 1.566 |
|  | Body weight | 0.120 | 0.043 | 7.788 | 0.005 | 1.127 | 1.036 | 1.226 |
|  | (constant) | -19.112 | 9.489 | 4.057 | 0.044 | 0.000 |  |  |

Binary logistic regression comparing hinge fractures group vs. no hinge fractures group.

Regression equation:

y(fracture or no fracture)=0.102* Post-op mLDFA +0.017* MAD correction +0.161* mLDFA correction +0.120* Body weight -19.112

y(fracture:1 no fracure:0)
