# Supplementary material for: Analysis of Intraoperative and Postoperative Hinge Fractures of Patients With Genu Valgum Treated With Lateral Open Wedge Distal Femoral Osteotomy
Source: Orthop Surg. 2025 Aug 5;17(9):2629–39. doi: 10.1111/os.70142 (PMC12404878; doi:10.1111/os.70142)
Supplement: Supplementary file 2 — Data S2: Supporting Information. [file OS-17-2629-s001.docx]

|  | | B | S.E | t | sig | tolerance | VIF |
| --- | --- | --- | --- | --- | --- | --- | --- |
|  | (constant) | 24.958 | 13.643 | 1.829 | 0.071 |  |  |
|  | mLDFA correction(degree) | -0.052 | 0.141 | -0.369 | 0.713 | 0.006 | 165.881 |
|  | mLDFA correction ratio | 7.509 | 11.024 | 0.681 | 0.498 | 0.006 | 164.853 |
|  | height(m) | -16.866 | 8.594 | -1.963 | 0.053 | 0.004 | 232.374 |
|  | body weight(kg) | 0.244 | 0.115 | 2.113 | 0.038 | 0.002 | 412.072 |
|  | BMI | -0.557 | 0.300 | -1.859 | 0.067 | 0.006 | 153.926 |
|  | sex | 0.157 | 0.200 | 0.786 | 0.434 | 0.359 | 2.786 |

Collinearity statistics show the mLDFA correction, mLDFA correction ratio, hight, body weight, BMI’ VIF are more than 10 which means these independent variables have collinearity. We eliminated mLDFA correction ratio, height, body weight. We use mLDFA correction, BMI, sex to do collinearity statistics again.

|  | | B | S.E. | t | sig | tolerance | VIF |
| --- | --- | --- | --- | --- | --- | --- | --- |
|  | (constant) | -1.248 | 0.751 | -1.663 | 0.100 |  |  |
|  | mLDFA correction | 0.040 | 0.012 | 3.412 | 0.001 | 0.918 | 1.090 |
|  | sex | -0.240 | 0.128 | -1.869 | 0.065 | 0.918 | 1.089 |
|  | BMI | 0.069 | 0.026 | 2.591 | 0.011 | 0.871 | 1.148 |

This time, all VIF are smaller than 10.We use mLDFA correction, sex and BMI to do binary logistic regression comparing intraoperative hinge fracture vs. no intraoperative hinge fracture.

|  | | | | | | | | | |
| --- | --- | --- | --- | --- | --- | --- | --- | --- | --- |
|  | | B | S.E, | Wals | df | Sig. | Exp (B) | EXP(B) 95% C.I. | |
|  |  |  |  |  |  |  |  | lower | upper |
|  | mLDFA correction | 0.247 | 0.083 | 8.797 | 1 | 0.003 | 1.280 | 1.087 | 1.506 |
|  | sex | -1.236 | 0.763 | 2.624 | 1 | 0.105 | 0.291 | 0.065 | 1.296 |
|  | BMI | 0.440 | 0.183 | 5.808 | 1 | 0.016 | 1.553 | 1.086 | 2.222 |
|  | constant | -11.465 | 5.009 | 5.240 | 1 | 0.022 | 0.000 |  |  |
| Binary logistic regression comparing intraoperative hinge fracture vs. no intraoperative hinge fracture | | | | | | | | | |

Regression equation:

y(IHF)=0.247*mLDFA correction-1.236*sex+0.440*BMI-11.465

y(IHF:1,no IHF:0)

Sex (Male:1 Female:2)
