# Supplementary material for: Analysis of Intraoperative and Postoperative Hinge Fractures of Patients With Genu Valgum Treated With Lateral Open Wedge Distal Femoral Osteotomy
Source: Orthop Surg. 2025 Aug 5;17(9):2629–39. doi: 10.1111/os.70142 (PMC12404878; doi:10.1111/os.70142)
Supplement: Supplementary file 3 — Data S3: Supporting Information. [file OS-17-2629-s002.docx]

| **group statistic** | | | | | |
| --- | --- | --- | --- | --- | --- |
|  | IHF | N | mean | S.E. | Standard error of the mean |
| age | 1 | 20 | 60.00 | 9.437 | 2.110 |
|  | 0 | 67 | 58.24 | 9.689 | 1.184 |
| Actual opening distance (mm) | 1 | 20 | 12.96300 | 4.590056 | 1.026368 |
|  | 0 | 67 | 11.62552 | 3.583167 | .437754 |
| Actual opening degree(°) | 1 | 20 | 12.63200 | 5.021782 | 1.122905 |
|  | 0 | 67 | 11.55006 | 5.143761 | .628410 |
| Planned opening distance (mm) | 1 | 20 | 11.45050 | 5.171188 | 1.156313 |
|  | 0 | 67 | 9.26761 | 4.046086 | .494308 |
| Planned opening degree (°) | 1 | 20 | 10.73550 | 4.328418 | .967864 |
|  | 0 | 67 | 9.22851 | 4.249758 | .519191 |
| Actual planned opening distance spread（mm） | 1 | 20 | 1.51250 | 3.586596 | .801987 |
|  | 0 | 67 | 2.35791 | 2.535863 | .309805 |
| Actual planned opening degree spread（°） | 1 | 20 | 1.89650 | 4.042877 | .904015 |
|  | 0 | 67 | 2.32155 | 3.236738 | .395430 |
| insall index | 1 | 20 | 1.19465 | .269036 | .060158 |
|  | 0 | 67 | 1.17496 | .227753 | .027824 |
| pre-op WOMAC | 1 | 20 | 28.55 | 15.988 | 3.575 |
|  | 0 | 67 | 25.84 | 13.197 | 1.612 |
| post-op WOMAC | 1 | 20 | 11.45 | 12.185 | 2.725 |
|  | 0 | 67 | 9.18 | 6.578 | .804 |
| Pre- and post-operative WOMAC difference | 1 | 20 | 17.10 | 5.902 | 1.320 |
|  | 0 | 67 | 16.66 | 8.538 | 1.043 |
| Postoperative lengthening of the affected limb | 1 | 20 | 3.6000 | 4.29688 | .96081 |
|  | 0 | 67 | 7.2090 | 8.56267 | 1.04610 |
| Healing time（months） | 1 | 20 | 3.900 | 1.3917 | .3112 |
|  | 0 | 67 | 2.799 | .6517 | .0796 |
| Pre-op MAD（mm） | 1 | 20 | -37.47350 | 23.951272 | 5.355667 |
|  | 0 | 67 | -28.47507 | 15.393072 | 1.880563 |
| Post-op MAD（mm） | 1 | 20 | .56250 | 8.205624 | 1.834833 |
|  | 0 | 67 | -1.82597 | 10.164771 | 1.241824 |
| Pre-op mLDFA （°） | 1 | 20 | 82.34350 | 2.917889 | .652460 |
|  | 0 | 67 | 83.59537 | 2.991314 | .365447 |
| Post-op mLDFA （°） | 1 | 20 | 93.01900 | 3.555352 | .795001 |
|  | 0 | 67 | 91.69940 | 3.251222 | .397200 |
| MAD correction (mm） | 1 | 20 | 38.03600 | 24.671839 | 5.516791 |
|  | 0 | 67 | 26.64910 | 13.728035 | 1.677146 |
| mLDFA correction (°) | 1 | 20 | 10.67550 | 4.321835 | .966392 |
|  | 0 | 67 | 8.10403 | 3.252861 | .397400 |
| WOMAC improvement ratio | 1 | 20 | .65584 | .153345 | .034289 |
|  | 0 | 67 | .67648 | .219489 | .026815 |
| MAD correction ratio | 1 | 20 | 1.02874 | .307999 | .068871 |
|  | 0 | 67 | .98307 | .341716 | .041747 |
| mLDFA correction ratio | 1 | 20 | .13084 | .056108 | .012546 |
|  | 0 | 67 | .09772 | .041022 | .005012 |
| Height | 1 | 20 | 1.6710 | .07677 | .01717 |
|  | 0 | 67 | 1.6322 | .06746 | .00824 |
| Body weight | 1 | 20 | 66.4500 | 8.17232 | 1.82739 |
|  | 0 | 67 | 60.8657 | 6.20092 | .75756 |
| BMI | 1 | 20 | 23.72935 | 1.721174 | .384866 |
|  | 0 | 67 | 22.82142 | 1.597093 | .195116 |

| **independent sample test** | | | | | | | | | | |
| --- | --- | --- | --- | --- | --- | --- | --- | --- | --- | --- |
|  | | Levene’s Test for Equality of Variances | | t-test for Equality of Means | | | | | | |
|  |  | F | Sig. | t | df | Sig.(2-tailed) | Mean Difference | standard error value | 95% Confidence Interval of the Difference | |
|  |  |  |  |  |  |  |  |  | Lower | Upper |
| age | Equal variances assumed | .003 | .955 | .717 | 85 | .475 | 1.761 | 2.455 | -3.119 | 6.642 |
|  | Equal variances not assumed |  |  | .728 | 31.930 | .472 | 1.761 | 2.419 | -3.168 | 6.690 |
| Actual opening distance (mm) | Equal variances assumed | .955 | .331 | 1.370 | 85 | .174 | 1.337478 | .976225 | -.603519 | 3.278475 |
|  | Equal variances not assumed |  |  | 1.199 | 26.291 | .241 | 1.337478 | 1.115822 | -.954893 | 3.629848 |
| Actual opening degree(°) | Equal variances assumed | .090 | .765 | .830 | 85 | .409 | 1.081940 | 1.303771 | -1.510305 | 3.674185 |
|  | Equal variances not assumed |  |  | .841 | 31.865 | .407 | 1.081940 | 1.286784 | -1.539590 | 3.703470 |
| Planned opening distance (mm) | Equal variances assumed | 2.865 | .094 | 1.982 | 85 | .051 | 2.182888 | 1.101537 | -.007262 | 4.373038 |
|  | Equal variances not assumed |  |  | 1.736 | 26.326 | .094 | 2.182888 | 1.257537 | -.400461 | 4.766237 |
| Planned opening degree (°) | Equal variances assumed | .181 | .671 | 1.386 | 85 | .169 | 1.506993 | 1.087370 | -.654990 | 3.668975 |
|  | Equal variances not assumed |  |  | 1.372 | 30.774 | .180 | 1.506993 | 1.098326 | -.733723 | 3.747708 |
| Actual planned opening spread | Equal variances assumed | 1.626 | .206 | -1.183 | 85 | .240 | -.845410 | .714752 | -2.266529 | .575708 |
|  | Equal variances not assumed |  |  | -.983 | 24.934 | .335 | -.845410 | .859746 | -2.616328 | .925507 |
| Actual planned opening degree spread（°） | Equal variances assumed | 1.814 | .182 | -.486 | 85 | .628 | -.425052 | .874846 | -2.164480 | 1.314376 |
|  | Equal variances not assumed |  |  | -.431 | 26.685 | .670 | -.425052 | .986716 | -2.450745 | 1.600640 |
| insall index | Equal variances assumed | 2.583 | .112 | .325 | 85 | .746 | .019695 | .060543 | -.100680 | .140070 |
|  | Equal variances not assumed |  |  | .297 | 27.635 | .769 | .019695 | .066281 | -.116157 | .155547 |
| pre-op WOMAC | Equal variances assumed | .044 | .834 | .768 | 85 | .445 | 2.714 | 3.534 | -4.313 | 9.741 |
|  | Equal variances not assumed |  |  | .692 | 27.191 | .495 | 2.714 | 3.922 | -5.330 | 10.759 |
| post-op WOMAC | Equal variances assumed | 2.518 | .116 | 1.091 | 85 | .279 | 2.271 | 2.082 | -1.869 | 6.411 |
|  | Equal variances not assumed |  |  | .799 | 22.401 | .432 | 2.271 | 2.841 | -3.614 | 8.156 |
| Pre- and post-operative WOMAC difference | Equal variances assumed | 2.712 | .103 | .217 | 85 | .829 | .443 | 2.045 | -3.622 | 4.508 |
|  | Equal variances not assumed |  |  | .264 | 45.089 | .793 | .443 | 1.682 | -2.945 | 3.831 |
| Postoperative lengthening of the affected limb | Equal variances assumed | 3.125 | .081 | -1.813 | 85 | .073 | -3.60896 | 1.99102 | -7.56764 | .34973 |
|  | Equal variances not assumed |  |  | -2.541 | 64.608 | .013 | -3.60896 | 1.42038 | -6.44597 | -.77194 |
| Healing time（months） | Equal variances assumed | 2.273 | .135 | 4.950 | 85 | .000 | 1.1015 | .2225 | .6590 | 1.5439 |
|  | Equal variances not assumed |  |  | 3.429 | 21.542 | .002 | 1.1015 | .3212 | .4345 | 1.7685 |
| Pre-op MAD（mm） | Equal variances assumed | 6.509 | .013 | -1.999 | 85 | .049 | -8.998425 | 4.502281 | -17.950165 | -.046685 |
|  | Equal variances not assumed |  |  | -1.585 | 23.870 | .126 | -8.998425 | 5.676239 | -20.716994 | 2.720143 |
| Post-op MAD（mm） | Equal variances assumed | .015 | .904 | .960 | 85 | .340 | 2.388470 | 2.487155 | -2.556660 | 7.333600 |
|  | Equal variances not assumed |  |  | 1.078 | 38.092 | .288 | 2.388470 | 2.215568 | -2.096356 | 6.873296 |
| Pre-op mLDFA （°） | Equal variances assumed | .031 | .861 | -1.651 | 85 | .102 | -1.251873 | .758059 | -2.759097 | .255350 |
|  | Equal variances not assumed |  |  | -1.674 | 31.888 | .104 | -1.251873 | .747834 | -2.775371 | .271625 |
| Post-op mLDFA （°） | Equal variances assumed | .086 | .770 | 1.559 | 85 | .123 | 1.319597 | .846364 | -.363202 | 3.002396 |
|  | Equal variances not assumed |  |  | 1.485 | 29.147 | .148 | 1.319597 | .888704 | -.497609 | 3.136803 |
| MAD correction (mm） | Equal variances assumed | 8.122 | .005 | 2.659 | 85 | .009 | 11.386896 | 4.281893 | 2.873345 | 19.900446 |
|  | Equal variances not assumed |  |  | 1.975 | 22.619 | .061 | 11.386896 | 5.766091 | -.552310 | 23.326101 |
| mLDFA correction (°) | Equal variances assumed | 1.186 | .279 | 2.867 | 85 | .005 | 2.571470 | .896936 | .788121 | 4.354819 |
|  | Equal variances not assumed |  |  | 2.461 | 25.757 | .021 | 2.571470 | 1.044911 | .422638 | 4.720302 |
| WOMAC improvement ratio | Equal variances assumed | .401 | .528 | -.392 | 85 | .696 | -.020645 | .052630 | -.125287 | .083997 |
|  | Equal variances not assumed |  |  | -.474 | 44.549 | .638 | -.020645 | .043529 | -.108341 | .067051 |
| MAD correction ratio | Equal variances assumed | .001 | .970 | .536 | 85 | .593 | .045674 | .085226 | -.123778 | .215125 |
|  | Equal variances not assumed |  |  | .567 | 34.199 | .574 | .045674 | .080536 | -.117960 | .209307 |
| mLDFA correction ratio | Equal variances assumed | 1.704 | .195 | 2.899 | 85 | .005 | .033121 | .011425 | .010406 | .055836 |
|  | Equal variances not assumed |  |  | 2.452 | 25.361 | .021 | .033121 | .013510 | .005316 | .060925 |
| Height | Equal variances assumed | .689 | .409 | 2.184 | 85 | .032 | .03876 | .01775 | .00348 | .07405 |
|  | Equal variances not assumed |  |  | 2.036 | 28.335 | .051 | .03876 | .01904 | -.00022 | .07775 |
| Body weight | Equal variances assumed | 2.747 | .101 | 3.275 | 85 | .002 | 5.58433 | 1.70520 | 2.19394 | 8.97472 |
|  | Equal variances not assumed |  |  | 2.823 | 25.872 | .009 | 5.58433 | 1.97819 | 1.51712 | 9.65154 |
| BMI | Equal variances assumed | .185 | .668 | 2.192 | 85 | .031 | .907931 | .414223 | .084344 | 1.731518 |
|  | Equal variances not assumed |  |  | 2.104 | 29.462 | .044 | .907931 | .431500 | .026014 | 1.789848 |

| **group statistic** | | | | | |
| --- | --- | --- | --- | --- | --- |
|  | PHF | N | mean | S.E. | Standard error of the mean |
| age | 1 | 16 | 59.19 | 10.968 | 2.742 |
|  | 0 | 71 | 58.52 | 9.355 | 1.110 |
| Actual opening distance (mm) | 1 | 16 | 12.24250 | 2.835357 | .708839 |
|  | 0 | 71 | 11.86324 | 4.058094 | .481607 |
| Actual opening degree(°) | 1 | 16 | 11.46525 | 5.031161 | 1.257790 |
|  | 0 | 71 | 11.87394 | 5.156901 | .612012 |
| Planned opening distance (mm) | 1 | 16 | 9.60875 | 3.248895 | .812224 |
|  | 0 | 71 | 9.80563 | 4.633900 | .549943 |
| Planned opening degree (°) | 1 | 16 | 9.35437 | 3.705229 | .926307 |
|  | 0 | 71 | 9.62465 | 4.433722 | .526186 |
| Actual planned opening spread | 1 | 16 | 2.63375 | 2.367600 | .591900 |
|  | 0 | 71 | 2.05761 | 2.906693 | .344961 |
| Actual planned opening degree spread（°） | 1 | 16 | 2.11088 | 3.744243 | .936061 |
|  | 0 | 71 | 2.24930 | 3.368428 | .399759 |
| insall index | 1 | 16 | 1.26163 | .242535 | .060634 |
|  | 0 | 71 | 1.16097 | .232688 | .027615 |
| pre-op WOMAC | 1 | 16 | 24.94 | 12.907 | 3.227 |
|  | 0 | 71 | 26.80 | 14.102 | 1.674 |
| post-op WOMAC | 1 | 16 | 9.75 | 6.914 | 1.728 |
|  | 0 | 71 | 9.69 | 8.485 | 1.007 |
| Pre- and post-operative WOMAC difference | 1 | 16 | 15.19 | 8.479 | 2.120 |
|  | 0 | 71 | 17.11 | 7.882 | .935 |
| Postoperative lengthening of the affected limb | 1 | 16 | 8.7500 | 12.05266 | 3.01317 |
|  | 0 | 71 | 5.8451 | 6.65614 | .78994 |
| Healing time（months） | 1 | 16 | 2.688 | .4031 | .1008 |
|  | 0 | 71 | 3.134 | 1.0588 | .1257 |
| Pre-op MAD（mm） | 1 | 16 | -30.15938 | 12.494779 | 3.123695 |
|  | 0 | 71 | -30.63028 | 19.064008 | 2.262481 |
| Post-op MAD（mm） | 1 | 16 | .91063 | 7.884989 | 1.971247 |
|  | 0 | 71 | -1.76986 | 10.113679 | 1.200273 |
| Pre-op mLDFA （°） | 1 | 16 | 83.99125 | 2.693595 | .673399 |
|  | 0 | 71 | 83.15352 | 3.067050 | .363992 |
| Post-op mLDFA （°） | 1 | 16 | 93.47188 | 3.151434 | .787858 |
|  | 0 | 71 | 91.67169 | 3.323836 | .394467 |
| MAD correction (mm） | 1 | 16 | 31.07000 | 12.965540 | 3.241385 |
|  | 0 | 71 | 28.86042 | 18.289474 | 2.170561 |
| mLDFA correction (°) | 1 | 16 | 9.48063 | 3.212522 | .803131 |
|  | 0 | 71 | 8.51817 | 3.757254 | .445904 |
| WOMAC improvement ratio | 1 | 16 | .64086 | .203958 | .050990 |
|  | 0 | 71 | .67870 | .206682 | .024529 |
| MAD correction ratio | 1 | 16 | 1.05101 | .326703 | .081676 |
|  | 0 | 71 | .98062 | .335424 | .039807 |
| mLDFA correction ratio | 1 | 16 | .11352 | .040298 | .010075 |
|  | 0 | 71 | .10349 | .048122 | .005711 |
| Height | 1 | 16 | 1.6394 | .06962 | .01740 |
|  | 0 | 71 | 1.6415 | .07199 | .00854 |
| Body weight | 1 | 16 | 61.8750 | 5.81808 | 1.45452 |
|  | 0 | 71 | 62.2113 | 7.34636 | .87185 |
| BMI | 1 | 16 | 23.01977 | 1.654159 | .413540 |
|  | 0 | 71 | 23.03247 | 1.674532 | .198730 |

| **independent sample test** | | | | | | | | | | |
| --- | --- | --- | --- | --- | --- | --- | --- | --- | --- | --- |
|  | | Levene’s Test for Equality of Variances | | t-test for Equality of Means | | | | | | |
|  |  | F | Sig. | t | df | Sig.(2-tailed) | Mean Difference | standard error value | 95% Confidence Interval of the Difference | |
|  |  |  |  |  |  |  |  |  | Lower | Upper |
| age | Equal variances assumed | .840 | .362 | .249 | 85 | .804 | .666 | 2.673 | -4.648 | 5.981 |
|  | Equal variances not assumed |  |  | .225 | 20.205 | .824 | .666 | 2.958 | -5.500 | 6.833 |
| Actual opening distance (mm) | Equal variances assumed | 1.264 | .264 | .354 | 85 | .724 | .379261 | 1.071115 | -1.750403 | 2.508925 |
|  | Equal variances not assumed |  |  | .443 | 30.646 | .661 | .379261 | .856970 | -1.369361 | 2.127882 |
| Actual opening degree(°) | Equal variances assumed | .113 | .738 | -.288 | 85 | .774 | -.408694 | 1.421037 | -3.234096 | 2.416709 |
|  | Equal variances not assumed |  |  | -.292 | 22.671 | .773 | -.408694 | 1.398783 | -3.304621 | 2.487234 |
| Planned opening distance (mm) | Equal variances assumed | 1.532 | .219 | -.161 | 85 | .873 | -.196884 | 1.223499 | -2.629527 | 2.235760 |
|  | Equal variances not assumed |  |  | -.201 | 30.531 | .842 | -.196884 | .980890 | -2.198668 | 1.804901 |
| Planned opening degree (°) | Equal variances assumed | .876 | .352 | -.226 | 85 | .821 | -.270273 | 1.193883 | -2.644033 | 2.103487 |
|  | Equal variances not assumed |  |  | -.254 | 25.669 | .802 | -.270273 | 1.065325 | -2.461453 | 1.920907 |
| Actual planned opening spread | Equal variances assumed | .202 | .655 | .739 | 85 | .462 | .576144 | .780144 | -.974991 | 2.127280 |
|  | Equal variances not assumed |  |  | .841 | 26.271 | .408 | .576144 | .685087 | -.831365 | 1.983654 |
| Actual planned opening degree spread（°） | Equal variances assumed | .503 | .480 | -.145 | 85 | .885 | -.138421 | .951356 | -2.029971 | 1.753129 |
|  | Equal variances not assumed |  |  | -.136 | 20.822 | .893 | -.138421 | 1.017849 | -2.256256 | 1.979414 |
| insall index | Equal variances assumed | .002 | .963 | 1.551 | 85 | .125 | .100653 | .064883 | -.028352 | .229658 |
|  | Equal variances not assumed |  |  | 1.511 | 21.668 | .145 | .100653 | .066626 | -.037644 | .238950 |
| pre-op WOMAC | Equal variances assumed | .495 | .484 | -.485 | 85 | .629 | -1.865 | 3.846 | -9.513 | 5.782 |
|  | Equal variances not assumed |  |  | -.513 | 23.787 | .613 | -1.865 | 3.635 | -9.371 | 5.641 |
| post-op WOMAC | Equal variances assumed | .120 | .730 | .026 | 85 | .979 | .060 | 2.277 | -4.468 | 4.588 |
|  | Equal variances not assumed |  |  | .030 | 26.261 | .976 | .060 | 2.000 | -4.050 | 4.170 |
| Pre- and post-operative WOMAC difference | Equal variances assumed | .019 | .890 | -.871 | 85 | .386 | -1.925 | 2.211 | -6.322 | 2.472 |
|  | Equal variances not assumed |  |  | -.831 | 21.239 | .415 | -1.925 | 2.317 | -6.740 | 2.890 |
| Postoperative lengthening of the affected limb | Equal variances assumed | 5.611 | .020 | 1.332 | 85 | .186 | 2.90493 | 2.18117 | -1.43183 | 7.24169 |
|  | Equal variances not assumed |  |  | .933 | 17.115 | .364 | 2.90493 | 3.11499 | -3.66375 | 9.47361 |
| Healing time（months） | Equal variances assumed | 3.316 | .072 | -1.653 | 85 | .102 | -.4463 | .2700 | -.9832 | .0905 |
|  | Equal variances not assumed |  |  | -2.771 | 64.495 | .007 | -.4463 | .1611 | -.7680 | -.1246 |
| Pre-op MAD（mm） | Equal variances assumed | .911 | .343 | .094 | 85 | .925 | .470907 | 5.003172 | -9.476739 | 10.418552 |
|  | Equal variances not assumed |  |  | .122 | 32.925 | .904 | .470907 | 3.856980 | -7.376860 | 8.318673 |
| Post-op MAD（mm） | Equal variances assumed | .488 | .487 | .993 | 85 | .324 | 2.680484 | 2.700265 | -2.688366 | 8.049335 |
|  | Equal variances not assumed |  |  | 1.161 | 27.378 | .255 | 2.680484 | 2.307915 | -2.051910 | 7.412878 |
| Pre-op mLDFA （°） | Equal variances assumed | .013 | .909 | 1.008 | 85 | .317 | .837729 | .831468 | -.815453 | 2.490911 |
|  | Equal variances not assumed |  |  | 1.094 | 24.596 | .284 | .837729 | .765477 | -.740117 | 2.415574 |
| Post-op mLDFA （°） | Equal variances assumed | .096 | .757 | 1.975 | 85 | .052 | 1.800185 | .911597 | -.012315 | 3.612684 |
|  | Equal variances not assumed |  |  | 2.043 | 23.151 | .053 | 1.800185 | .881093 | -.021836 | 3.622206 |
| MAD correction (mm） | Equal variances assumed | .957 | .331 | .457 | 85 | .649 | 2.209577 | 4.834151 | -7.402009 | 11.821164 |
|  | Equal variances not assumed |  |  | .566 | 30.169 | .575 | 2.209577 | 3.901014 | -5.755488 | 10.174643 |
| mLDFA correction (°) | Equal variances assumed | .351 | .555 | .948 | 85 | .346 | .962456 | 1.014805 | -1.055248 | 2.980160 |
|  | Equal variances not assumed |  |  | 1.048 | 25.161 | .305 | .962456 | .918613 | -.928850 | 2.853762 |
| WOMAC improvement ratio | Equal variances assumed | .155 | .695 | -.663 | 85 | .509 | -.037842 | .057065 | -.151302 | .075618 |
|  | Equal variances not assumed |  |  | -.669 | 22.488 | .510 | -.037842 | .056583 | -.155040 | .079356 |
| MAD correction ratio | Equal variances assumed | .001 | .981 | .762 | 85 | .448 | .070390 | .092404 | -.113333 | .254113 |
|  | Equal variances not assumed |  |  | .775 | 22.698 | .447 | .070390 | .090860 | -.117707 | .258487 |
| mLDFA correction ratio | Equal variances assumed | .552 | .460 | .774 | 85 | .441 | .010029 | .012961 | -.015742 | .035800 |
|  | Equal variances not assumed |  |  | .866 | 25.622 | .395 | .010029 | .011581 | -.013792 | .033851 |
| Height | Equal variances assumed | .003 | .958 | -.110 | 85 | .913 | -.00217 | .01981 | -.04156 | .03721 |
|  | Equal variances not assumed |  |  | -.112 | 22.816 | .912 | -.00217 | .01939 | -.04230 | .03795 |
| Body weight | Equal variances assumed | 1.714 | .194 | -.171 | 85 | .865 | -.33627 | 1.96501 | -4.24324 | 3.57071 |
|  | Equal variances not assumed |  |  | -.198 | 26.969 | .844 | -.33627 | 1.69580 | -3.81596 | 3.14342 |
| BMI | Equal variances assumed | .000 | .988 | -.027 | 85 | .978 | -.012698 | .462418 | -.932109 | .906714 |
|  | Equal variances not assumed |  |  | -.028 | 22.471 | .978 | -.012698 | .458812 | -.963061 | .937665 |

| **group statistic** | | | | | |
| --- | --- | --- | --- | --- | --- |
|  | fracture or not | N | mean | S.E. | Standard error of the mean |
| age | 1 | 36 | 59.64 | 10.003 | 1.667 |
|  | 0 | 51 | 57.94 | 9.352 | 1.310 |
| Actual opening distance (mm) | 1 | 36 | 12.64278 | 3.874852 | .645809 |
|  | 0 | 51 | 11.43196 | 3.791543 | .530922 |
| Actual opening degree(°) | 1 | 36 | 12.11344 | 4.988377 | .831396 |
|  | 0 | 51 | 11.57667 | 5.227631 | .732015 |
| Planned opening distance (mm) | 1 | 36 | 10.63194 | 4.461146 | .743524 |
|  | 0 | 51 | 9.16059 | 4.288817 | .600555 |
| Planned opening degree (°) | 1 | 36 | 10.12167 | 4.066783 | .677797 |
|  | 0 | 51 | 9.18902 | 4.440102 | .621739 |
| Actual planned opening spread | 1 | 36 | 2.01083 | 3.115252 | .519209 |
|  | 0 | 51 | 2.27137 | 2.602835 | .364470 |
| Actual planned opening degree spread（°） | 1 | 36 | 1.99178 | 3.859130 | .643188 |
|  | 0 | 51 | 2.38765 | 3.099109 | .433962 |
| insall index | 1 | 36 | 1.22442 | .256206 | .042701 |
|  | 0 | 51 | 1.14776 | .218324 | .030571 |
| pre-op WOMAC | 1 | 36 | 26.94 | 14.611 | 2.435 |
|  | 0 | 51 | 26.12 | 13.401 | 1.877 |
| post-op WOMAC | 1 | 36 | 10.69 | 10.091 | 1.682 |
|  | 0 | 51 | 9.00 | 6.530 | .914 |
| Pre- and post-operative WOMAC difference | 1 | 36 | 16.25 | 7.117 | 1.186 |
|  | 0 | 51 | 17.12 | 8.588 | 1.202 |
| Postoperative lengthening of the affected limb | 1 | 36 | 5.8889 | 8.88909 | 1.48151 |
|  | 0 | 51 | 6.7255 | 7.22517 | 1.01173 |
| Healing time（months） | 1 | 36 | 3.361 | 1.2225 | .2037 |
|  | 0 | 51 | 2.833 | .7118 | .0997 |
| Pre-op MAD（mm） | 1 | 36 | -34.22278 | 19.796769 | 3.299461 |
|  | 0 | 51 | -27.94667 | 16.270869 | 2.278377 |
| Post-op MAD（mm） | 1 | 36 | .71722 | 7.951614 | 1.325269 |
|  | 0 | 51 | -2.68451 | 10.704465 | 1.498925 |
| Pre-op mLDFA （°） | 1 | 36 | 83.07583 | 2.901887 | .483648 |
|  | 0 | 51 | 83.47118 | 3.093346 | .433155 |
| Post-op mLDFA （°） | 1 | 36 | 93.22028 | 3.342224 | .557037 |
|  | 0 | 51 | 91.14333 | 3.106895 | .435052 |
| MAD correction (mm） | 1 | 36 | 34.94000 | 20.366784 | 3.394464 |
|  | 0 | 51 | 25.26216 | 13.788327 | 1.930752 |
| mLDFA correction (°) | 1 | 36 | 10.14444 | 3.863316 | .643886 |
|  | 0 | 51 | 7.67216 | 3.174006 | .444450 |
| WOMAC improvement ratio | 1 | 36 | .64918 | .175072 | .029179 |
|  | 0 | 51 | .68766 | .224896 | .031492 |
| MAD correction ratio | 1 | 36 | 1.03864 | .312037 | .052006 |
|  | 0 | 51 | .96175 | .346663 | .048542 |
| mLDFA correction ratio | 1 | 36 | .12314 | .049811 | .008302 |
|  | 0 | 51 | .09276 | .040363 | .005652 |
| Height | 1 | 36 | 1.6569 | .07437 | .01239 |
|  | 0 | 51 | 1.6300 | .06732 | .00943 |
| Body weight | 1 | 36 | 64.4167 | 7.48856 | 1.24809 |
|  | 0 | 51 | 60.5490 | 6.33818 | .88752 |
| BMI | 1 | 36 | 23.41398 | 1.705500 | .284250 |
|  | 0 | 51 | 22.75919 | 1.590446 | .222707 |

| **independent sample test** | | | | | | | | | | |
| --- | --- | --- | --- | --- | --- | --- | --- | --- | --- | --- |
|  | | Levene’s Test for Equality of Variances | | t-test for Equality of Means | | | | | | |
|  |  | F | Sig. | t | df | Sig.(2-tailed) | Mean Difference | standard error value | 95% Confidence Interval of the Difference | |
|  |  |  |  |  |  |  |  |  | Lower | Upper |
| age | Equal variances assumed | .478 | .491 | .810 | 85 | .420 | 1.698 | 2.095 | -2.468 | 5.864 |
|  | Equal variances not assumed |  |  | .801 | 72.256 | .426 | 1.698 | 2.120 | -2.528 | 5.924 |
| Actual opening distance (mm) | Equal variances assumed | .029 | .865 | 1.454 | 85 | .150 | 1.210817 | .832867 | -.445146 | 2.866780 |
|  | Equal variances not assumed |  |  | 1.448 | 74.482 | .152 | 1.210817 | .836031 | -.454831 | 2.876465 |
| Actual opening degree(°) | Equal variances assumed | .302 | .584 | .481 | 85 | .632 | .536778 | 1.116812 | -1.683744 | 2.757299 |
|  | Equal variances not assumed |  |  | .485 | 77.638 | .629 | .536778 | 1.107730 | -1.668704 | 2.742260 |
| Planned opening distance (mm) | Equal variances assumed | .347 | .557 | 1.550 | 85 | .125 | 1.471356 | .949226 | -.415959 | 3.358672 |
|  | Equal variances not assumed |  |  | 1.539 | 73.628 | .128 | 1.471356 | .955769 | -.433215 | 3.375927 |
| Planned opening degree (°) | Equal variances assumed | .029 | .866 | .999 | 85 | .321 | .932647 | .933927 | -.924249 | 2.789544 |
|  | Equal variances not assumed |  |  | 1.014 | 79.353 | .314 | .932647 | .919765 | -.897973 | 2.763267 |
| Actual planned opening spread | Equal variances assumed | .521 | .472 | -.424 | 85 | .673 | -.260539 | .614976 | -1.483277 | .962199 |
|  | Equal variances not assumed |  |  | -.411 | 66.662 | .683 | -.260539 | .634363 | -1.526850 | 1.005772 |
| Actual planned opening degree spread（°） | Equal variances assumed | 2.982 | .088 | -.530 | 85 | .598 | -.395869 | .747195 | -1.881493 | 1.089754 |
|  | Equal variances not assumed |  |  | -.510 | 64.729 | .612 | -.395869 | .775896 | -1.945563 | 1.153825 |
| insall index | Equal variances assumed | 2.782 | .099 | 1.501 | 85 | .137 | .076652 | .051082 | -.024913 | .178217 |
|  | Equal variances not assumed |  |  | 1.460 | 67.637 | .149 | .076652 | .052516 | -.028153 | .181457 |
| pre-op WOMAC | Equal variances assumed | .116 | .734 | .273 | 85 | .786 | .827 | 3.028 | -5.194 | 6.848 |
|  | Equal variances not assumed |  |  | .269 | 71.307 | .789 | .827 | 3.074 | -5.303 | 6.956 |
| post-op WOMAC | Equal variances assumed | .760 | .386 | .951 | 85 | .344 | 1.694 | 1.782 | -1.848 | 5.237 |
|  | Equal variances not assumed |  |  | .885 | 55.365 | .380 | 1.694 | 1.914 | -2.141 | 5.530 |
| Pre- and post-operative WOMAC difference | Equal variances assumed | 1.880 | .174 | -.497 | 85 | .620 | -.868 | 1.745 | -4.337 | 2.601 |
|  | Equal variances not assumed |  |  | -.514 | 82.737 | .609 | -.868 | 1.689 | -4.227 | 2.492 |
| Postoperative lengthening of the affected limb | Equal variances assumed | .336 | .564 | -.483 | 85 | .630 | -.83660 | 1.73114 | -4.27857 | 2.60537 |
|  | Equal variances not assumed |  |  | -.466 | 65.313 | .643 | -.83660 | 1.79401 | -4.41916 | 2.74596 |
| Healing time（months） | Equal variances assumed | .550 | .460 | 2.537 | 85 | .013 | .5278 | .2080 | .1141 | .9414 |
|  | Equal variances not assumed |  |  | 2.327 | 51.685 | .024 | .5278 | .2268 | .0726 | .9830 |
| Pre-op MAD（mm） | Equal variances assumed | 2.178 | .144 | -1.619 | 85 | .109 | -6.276111 | 3.876370 | -13.983374 | 1.431152 |
|  | Equal variances not assumed |  |  | -1.565 | 65.855 | .122 | -6.276111 | 4.009669 | -14.282004 | 1.729782 |
| Post-op MAD（mm） | Equal variances assumed | .544 | .463 | 1.617 | 85 | .110 | 3.401732 | 2.104195 | -.781972 | 7.585436 |
|  | Equal variances not assumed |  |  | 1.700 | 84.745 | .093 | 3.401732 | 2.000778 | -.576524 | 7.379988 |
| Pre-op mLDFA （°） | Equal variances assumed | .015 | .903 | -.602 | 85 | .549 | -.395343 | .656526 | -1.700693 | .910007 |
|  | Equal variances not assumed |  |  | -.609 | 78.370 | .544 | -.395343 | .649260 | -1.687824 | .897138 |
| Post-op mLDFA （°） | Equal variances assumed | .815 | .369 | 2.976 | 85 | .004 | 2.076944 | .697865 | .689401 | 3.464488 |
|  | Equal variances not assumed |  |  | 2.939 | 71.975 | .004 | 2.076944 | .706796 | .667963 | 3.485926 |
| MAD correction (mm） | Equal variances assumed | 3.101 | .082 | 2.644 | 85 | .010 | 9.677843 | 3.659632 | 2.401514 | 16.954172 |
|  | Equal variances not assumed |  |  | 2.478 | 57.125 | .016 | 9.677843 | 3.905149 | 1.858287 | 17.497399 |
| mLDFA correction (°) | Equal variances assumed | .768 | .383 | 3.269 | 85 | .002 | 2.472288 | .756325 | .968511 | 3.976064 |
|  | Equal variances not assumed |  |  | 3.160 | 65.836 | .002 | 2.472288 | .782384 | .910135 | 4.034440 |
| WOMAC improvement ratio | Equal variances assumed | .053 | .818 | -.859 | 85 | .393 | -.038482 | .044809 | -.127574 | .050610 |
|  | Equal variances not assumed |  |  | -.896 | 84.126 | .373 | -.038482 | .042932 | -.123854 | .046890 |
| MAD correction ratio | Equal variances assumed | .029 | .864 | 1.061 | 85 | .292 | .076888 | .072454 | -.067170 | .220945 |
|  | Equal variances not assumed |  |  | 1.081 | 80.030 | .283 | .076888 | .071141 | -.064686 | .218461 |
| mLDFA correction ratio | Equal variances assumed | .942 | .334 | 3.136 | 85 | .002 | .030379 | .009686 | .011120 | .049637 |
|  | Equal variances not assumed |  |  | 3.025 | 65.164 | .004 | .030379 | .010043 | .010322 | .050435 |
| Height | Equal variances assumed | .612 | .436 | 1.761 | 85 | .082 | .02694 | .01530 | -.00349 | .05737 |
|  | Equal variances not assumed |  |  | 1.730 | 70.654 | .088 | .02694 | .01557 | -.00411 | .05800 |
| Body weight | Equal variances assumed | 1.461 | .230 | 2.599 | 85 | .011 | 3.86765 | 1.48794 | .90923 | 6.82606 |
|  | Equal variances not assumed |  |  | 2.525 | 67.300 | .014 | 3.86765 | 1.53148 | .81105 | 6.92425 |
| BMI | Equal variances assumed | .404 | .527 | 1.836 | 85 | .070 | .654795 | .356738 | -.054496 | 1.364085 |
|  | Equal variances not assumed |  |  | 1.813 | 72.132 | .074 | .654795 | .361104 | -.065031 | 1.374621 |

Intraoperative fracture type was not associated with any of the measures

| **group statistic** | | | | | |
| --- | --- | --- | --- | --- | --- |
|  | PHF type | N | mean | S.E. | Standard error of the mean |
| Healing time（months） | 1 | 12 | 2.750 | .4523 | .1306 |
|  | 2 | 4 | 2.500 | .0000 | .0000 |
| age | 1 | 12 | 58.83 | 10.539 | 3.042 |
|  | 2 | 4 | 60.25 | 13.865 | 6.933 |
| Actual opening distance (mm) | 1 | 12 | 12.18583 | 2.903704 | .838227 |
|  | 2 | 4 | 12.41250 | 3.037986 | 1.518993 |
| Actual opening degree(°) | 1 | 12 | 10.61450 | 5.344543 | 1.542837 |
|  | 2 | 4 | 14.01750 | 3.201139 | 1.600570 |
| Planned opening distance (mm) | 1 | 12 | 9.80667 | 3.083620 | .890165 |
|  | 2 | 4 | 9.01500 | 4.157471 | 2.078736 |
| Planned opening degree (°) | 1 | 12 | 9.47083 | 3.729681 | 1.076666 |
|  | 2 | 4 | 9.00500 | 4.173891 | 2.086946 |
| Actual planned opening spread | 1 | 12 | 2.37917 | 2.469448 | .712868 |
|  | 2 | 4 | 3.39750 | 2.151904 | 1.075952 |
| Actual planned opening degree spread（°） | 1 | 12 | 1.14367 | 3.141977 | .907011 |
|  | 2 | 4 | 5.01250 | 4.351033 | 2.175517 |
| insall index | 1 | 12 | 1.25767 | .241339 | .069669 |
|  | 2 | 4 | 1.27350 | .283375 | .141688 |
| pre-op WOMAC | 1 | 12 | 22.17 | 10.895 | 3.145 |
|  | 2 | 4 | 33.25 | 16.581 | 8.290 |
| post-op WOMAC | 1 | 12 | 8.08 | 6.529 | 1.885 |
|  | 2 | 4 | 14.75 | 6.185 | 3.092 |
| Pre- and post-operative WOMAC difference | 1 | 12 | 14.08 | 6.487 | 1.873 |
|  | 2 | 4 | 18.50 | 13.626 | 6.813 |
| Postoperative lengthening of the affected limb | 1 | 12 | 10.0833 | 12.87322 | 3.71618 |
|  | 2 | 4 | 4.7500 | 9.50000 | 4.75000 |
| Pre-op MAD（mm） | 1 | 12 | -30.04583 | 13.025059 | 3.760011 |
|  | 2 | 4 | -30.50000 | 12.583057 | 6.291529 |
| Post-op MAD（mm） | 1 | 12 | -.30333 | 8.249602 | 2.381455 |
|  | 2 | 4 | 4.55250 | 6.143915 | 3.071958 |
| Pre-op mLDFA （°） | 1 | 12 | 83.63000 | 2.713940 | .783447 |
|  | 2 | 4 | 85.07500 | 2.680019 | 1.340009 |
| Post-op mLDFA （°） | 1 | 12 | 92.37917 | 2.712102 | .782917 |
|  | 2 | 4 | 96.75000 | 1.892969 | .946485 |
| MAD correction (mm） | 1 | 12 | 29.74250 | 13.152868 | 3.796906 |
|  | 2 | 4 | 35.05250 | 13.341827 | 6.670914 |
| mLDFA correction (°) | 1 | 12 | 8.74917 | 3.056669 | .882384 |
|  | 2 | 4 | 11.67500 | 2.963528 | 1.481764 |
| WOMAC improvement ratio | 1 | 12 | .67791 | .197450 | .056999 |
|  | 2 | 4 | .52969 | .207544 | .103772 |
| MAD correction ratio | 1 | 12 | 1.00757 | .336756 | .097213 |
|  | 2 | 4 | 1.18134 | .296076 | .148038 |
| mLDFA correction ratio | 1 | 12 | .10536 | .038784 | .011196 |
|  | 2 | 4 | .13799 | .039239 | .019619 |
| Height | 1 | 12 | 1.6400 | .07324 | .02114 |
|  | 2 | 4 | 1.6375 | .06752 | .03376 |
| Body weight | 1 | 12 | 61.8333 | 6.13238 | 1.77027 |
|  | 2 | 4 | 62.0000 | 5.59762 | 2.79881 |
| BMI | 1 | 12 | 22.97725 | 1.628313 | .470054 |
|  | 2 | 4 | 23.14736 | 1.982538 | .991269 |

1: type 1 fracure

2: type 3 fracture

| **independent sample test** | | | | | | | | | | |
| --- | --- | --- | --- | --- | --- | --- | --- | --- | --- | --- |
|  | | Levene’s Test for Equality of Variances | | t-test for Equality of Means | | | | | | |
|  |  | F | Sig. | t | df | Sig.(2-tailed) | Mean Difference | standard error value | 95% Confidence Interval of the Difference | |
|  |  |  |  |  |  |  |  |  | Lower | Upper |
| Healing time（months） | Equal variances assumed | 10.500 | .006 | 1.080 | 14 | .298 | .2500 | .2315 | -.2464 | .7464 |
|  | Equal variances not assumed |  |  | 1.915 | 11.000 | .082 | .2500 | .1306 | -.0374 | .5374 |
| age | Equal variances assumed | .488 | .496 | -.216 | 14 | .832 | -1.417 | 6.544 | -15.451 | 12.618 |
|  | Equal variances not assumed |  |  | -.187 | 4.224 | .860 | -1.417 | 7.571 | -22.004 | 19.171 |
| Actual opening distance (mm) | Equal variances assumed | .102 | .754 | -.134 | 14 | .895 | -.226667 | 1.693366 | -3.858576 | 3.405243 |
|  | Equal variances not assumed |  |  | -.131 | 4.979 | .901 | -.226667 | 1.734925 | -4.691997 | 4.238664 |
| Actual opening degree(°) | Equal variances assumed | 1.453 | .248 | -1.187 | 14 | .255 | -3.403000 | 2.865839 | -9.549614 | 2.743614 |
|  | Equal variances not assumed |  |  | -1.531 | 9.037 | .160 | -3.403000 | 2.223099 | -8.428851 | 1.622851 |
| Planned opening distance (mm) | Equal variances assumed | .690 | .420 | .410 | 14 | .688 | .791667 | 1.930023 | -3.347821 | 4.931155 |
|  | Equal variances not assumed |  |  | .350 | 4.163 | .743 | .791667 | 2.261313 | -5.390996 | 6.974330 |
| Planned opening degree (°) | Equal variances assumed | .014 | .907 | .211 | 14 | .836 | .465833 | 2.210795 | -4.275850 | 5.207517 |
|  | Equal variances not assumed |  |  | .198 | 4.718 | .851 | .465833 | 2.348308 | -5.680709 | 6.612375 |
| Actual planned opening spread | Equal variances assumed | .325 | .578 | -.733 | 14 | .475 | -1.018333 | 1.388490 | -3.996348 | 1.959681 |
|  | Equal variances not assumed |  |  | -.789 | 5.902 | .461 | -1.018333 | 1.290680 | -4.189303 | 2.152637 |
| Actual planned opening degree spread（°） | Equal variances assumed | .316 | .583 | -1.950 | 14 | .072 | -3.868833 | 1.984384 | -8.124913 | .387246 |
|  | Equal variances not assumed |  |  | -1.641 | 4.100 | .174 | -3.868833 | 2.357020 | -10.350633 | 2.612966 |
| insall index | Equal variances assumed | .085 | .775 | -.109 | 14 | .915 | -.015833 | .144881 | -.326571 | .294905 |
|  | Equal variances not assumed |  |  | -.100 | 4.553 | .924 | -.015833 | .157890 | -.433972 | .402305 |
| pre-op WOMAC | Equal variances assumed | .901 | .359 | -1.556 | 14 | .142 | -11.083 | 7.122 | -26.359 | 4.192 |
|  | Equal variances not assumed |  |  | -1.250 | 3.904 | .281 | -11.083 | 8.867 | -35.943 | 13.777 |
| post-op WOMAC | Equal variances assumed | .002 | .967 | -1.788 | 14 | .095 | -6.667 | 3.728 | -14.662 | 1.329 |
|  | Equal variances not assumed |  |  | -1.841 | 5.438 | .120 | -6.667 | 3.621 | -15.755 | 2.421 |
| Pre- and post-operative WOMAC difference | Equal variances assumed | 2.775 | .118 | -.896 | 14 | .385 | -4.417 | 4.928 | -14.986 | 6.153 |
|  | Equal variances not assumed |  |  | -.625 | 3.465 | .571 | -4.417 | 7.066 | -25.288 | 16.455 |
| Postoperative lengthening of the affected limb | Equal variances assumed | .097 | .760 | .755 | 14 | .463 | 5.33333 | 7.06040 | -9.80971 | 20.47638 |
|  | Equal variances not assumed |  |  | .884 | 7.074 | .406 | 5.33333 | 6.03096 | -8.89758 | 19.56425 |
| Pre-op MAD（mm） | Equal variances assumed | .010 | .920 | .061 | 14 | .952 | .454167 | 7.466072 | -15.558965 | 16.467298 |
|  | Equal variances not assumed |  |  | .062 | 5.340 | .953 | .454167 | 7.329462 | -18.031757 | 18.940090 |
| Post-op MAD（mm） | Equal variances assumed | .819 | .381 | -1.072 | 14 | .302 | -4.855833 | 4.529949 | -14.571607 | 4.859941 |
|  | Equal variances not assumed |  |  | -1.249 | 7.000 | .252 | -4.855833 | 3.886933 | -14.047010 | 4.335344 |
| Pre-op mLDFA （°） | Equal variances assumed | .040 | .844 | -.925 | 14 | .371 | -1.445000 | 1.562718 | -4.796697 | 1.906697 |
|  | Equal variances not assumed |  |  | -.931 | 5.235 | .393 | -1.445000 | 1.552229 | -5.381924 | 2.491924 |
| Post-op mLDFA （°） | Equal variances assumed | 1.735 | .209 | -2.959 | 14 | .010 | -4.370833 | 1.477292 | -7.539310 | -1.202356 |
|  | Equal variances not assumed |  |  | -3.558 | 7.546 | .008 | -4.370833 | 1.228329 | -7.233278 | -1.508389 |
| MAD correction (mm） | Equal variances assumed | .302 | .591 | -.697 | 14 | .497 | -5.310000 | 7.617321 | -21.647529 | 11.027529 |
|  | Equal variances not assumed |  |  | -.692 | 5.112 | .519 | -5.310000 | 7.675779 | -24.911596 | 14.291596 |
| mLDFA correction (°) | Equal variances assumed | .159 | .696 | -1.669 | 14 | .117 | -2.925833 | 1.753384 | -6.686469 | .834802 |
|  | Equal variances not assumed |  |  | -1.697 | 5.322 | .147 | -2.925833 | 1.724595 | -7.279478 | 1.427811 |
| WOMAC improvement ratio | Equal variances assumed | .180 | .678 | 1.286 | 14 | .219 | .148224 | .115271 | -.099008 | .395456 |
|  | Equal variances not assumed |  |  | 1.252 | 4.960 | .266 | .148224 | .118395 | -.156858 | .453306 |
| MAD correction ratio | Equal variances assumed | .176 | .681 | -.916 | 14 | .375 | -.173773 | .189638 | -.580507 | .232961 |
|  | Equal variances not assumed |  |  | -.981 | 5.849 | .365 | -.173773 | .177104 | -.609864 | .262318 |
| mLDFA correction ratio | Equal variances assumed | .072 | .793 | -1.454 | 14 | .168 | -.032630 | .022448 | -.080777 | .015517 |
|  | Equal variances not assumed |  |  | -1.444 | 5.124 | .207 | -.032630 | .022589 | -.090278 | .025018 |
| Height | Equal variances assumed | .032 | .862 | .060 | 14 | .953 | .00250 | .04160 | -.08672 | .09172 |
|  | Equal variances not assumed |  |  | .063 | 5.581 | .952 | .00250 | .03983 | -.09677 | .10177 |
| Body weight | Equal variances assumed | .179 | .679 | -.048 | 14 | .962 | -.16667 | 3.47668 | -7.62340 | 7.29007 |
|  | Equal variances not assumed |  |  | -.050 | 5.635 | .962 | -.16667 | 3.31167 | -8.39914 | 8.06581 |
| BMI | Equal variances assumed | .811 | .383 | -.172 | 14 | .866 | -.170110 | .987503 | -2.288093 | 1.947874 |
|  | Equal variances not assumed |  |  | -.155 | 4.440 | .884 | -.170110 | 1.097071 | -3.100723 | 2.760504 |

Total fracture type is not related to any measure

| **group statistic** | | | | | |
| --- | --- | --- | --- | --- | --- |
|  | IHF type | N | mean | S.E. | Standard error of the mean |
| age | 1.00 | 20 | 60.00 | 9.437 | 2.110 |
|  | 2.00 | 16 | 59.19 | 10.968 | 2.742 |
| Actual opening distance (mm) | 1.00 | 20 | 12.96300 | 4.590056 | 1.026368 |
|  | 2.00 | 16 | 12.24250 | 2.835357 | .708839 |
| Actual opening degree(°) | 1.00 | 20 | 12.63200 | 5.021782 | 1.122905 |
|  | 2.00 | 16 | 11.46525 | 5.031161 | 1.257790 |
| Planned opening distance (mm) | 1.00 | 20 | 11.45050 | 5.171188 | 1.156313 |
|  | 2.00 | 16 | 9.60875 | 3.248895 | .812224 |
| Planned opening degree (°) | 1.00 | 20 | 10.73550 | 4.328418 | .967864 |
|  | 2.00 | 16 | 9.35437 | 3.705229 | .926307 |
| Actual planned opening spread | 1.00 | 20 | 1.51250 | 3.586596 | .801987 |
|  | 2.00 | 16 | 2.63375 | 2.367600 | .591900 |
| Actual planned opening degree spread（°） | 1.00 | 20 | 1.89650 | 4.042877 | .904015 |
|  | 2.00 | 16 | 2.11088 | 3.744243 | .936061 |
| insall index | 1.00 | 20 | 1.19465 | .269036 | .060158 |
|  | 2.00 | 16 | 1.26163 | .242535 | .060634 |
| pre-op WOMAC | 1.00 | 20 | 28.55 | 15.988 | 3.575 |
|  | 2.00 | 16 | 24.94 | 12.907 | 3.227 |
| post-op WOMAC | 1.00 | 20 | 11.45 | 12.185 | 2.725 |
|  | 2.00 | 16 | 9.75 | 6.914 | 1.728 |
| Pre- and post-operative WOMAC difference | 1.00 | 20 | 17.10 | 5.902 | 1.320 |
|  | 2.00 | 16 | 15.19 | 8.479 | 2.120 |
| Postoperative lengthening of the affected limb | 1.00 | 20 | 3.6000 | 4.29688 | .96081 |
|  | 2.00 | 16 | 8.7500 | 12.05266 | 3.01317 |
| Healing time（months） | 1.00 | 20 | 3.900 | 1.3917 | .3112 |
|  | 2.00 | 16 | 2.688 | .4031 | .1008 |
| Pre-op MAD（mm） | 1.00 | 20 | -37.47350 | 23.951272 | 5.355667 |
|  | 2.00 | 16 | -30.15938 | 12.494779 | 3.123695 |
| Post-op MAD（mm） | 1.00 | 20 | .56250 | 8.205624 | 1.834833 |
|  | 2.00 | 16 | .91063 | 7.884989 | 1.971247 |
| Pre-op mLDFA （°） | 1.00 | 20 | 82.34350 | 2.917889 | .652460 |
|  | 2.00 | 16 | 83.99125 | 2.693595 | .673399 |
| Post-op mLDFA （°） | 1.00 | 20 | 93.01900 | 3.555352 | .795001 |
|  | 2.00 | 16 | 93.47188 | 3.151434 | .787858 |
| MAD correction (mm） | 1.00 | 20 | 38.03600 | 24.671839 | 5.516791 |
|  | 2.00 | 16 | 31.07000 | 12.965540 | 3.241385 |
| mLDFA correction (°) | 1.00 | 20 | 10.67550 | 4.321835 | .966392 |
|  | 2.00 | 16 | 9.48063 | 3.212522 | .803131 |
| WOMAC improvement ratio | 1.00 | 20 | .65584 | .153345 | .034289 |
|  | 2.00 | 16 | .64086 | .203958 | .050990 |
| MAD correction ratio | 1.00 | 20 | 1.02874 | .307999 | .068871 |
|  | 2.00 | 16 | 1.05101 | .326703 | .081676 |
| mLDFA correction ratio | 1.00 | 20 | .13084 | .056108 | .012546 |
|  | 2.00 | 16 | .11352 | .040298 | .010075 |
| Height | 1.00 | 20 | 1.6710 | .07677 | .01717 |
|  | 2.00 | 16 | 1.6394 | .06962 | .01740 |
| Body weight | 1.00 | 20 | 66.4500 | 8.17232 | 1.82739 |
|  | 2.00 | 16 | 61.8750 | 5.81808 | 1.45452 |
| BMI | 1.00 | 20 | 23.72935 | 1.721174 | .384866 |
|  | 2.00 | 16 | 23.01977 | 1.654159 | .413540 |

1: type 1 fracure

2: type 3 fracure

| **independent sample test** | | | | | | | | | | |
| --- | --- | --- | --- | --- | --- | --- | --- | --- | --- | --- |
|  | | Levene’s Test for Equality of Variances | | t-test for Equality of Means | | | | | | |
|  |  | F | Sig. | t | df | Sig.(2-tailed) | Mean Difference | standard error value | 95% Confidence Interval of the Difference | |
|  |  |  |  |  |  |  |  |  | Lower | Upper |
| age | Equal variances assumed | .448 | .508 | .239 | 34 | .813 | .813 | 3.401 | -6.100 | 7.725 |
|  | Equal variances not assumed |  |  | .235 | 29.781 | .816 | .813 | 3.460 | -6.256 | 7.881 |
| Actual opening distance (mm) | Equal variances assumed | 1.586 | .216 | .549 | 34 | .587 | .720500 | 1.312837 | -1.947506 | 3.388506 |
|  | Equal variances not assumed |  |  | .578 | 32.175 | .568 | .720500 | 1.247351 | -1.819727 | 3.260727 |
| Actual opening degree(°) | Equal variances assumed | .001 | .974 | .692 | 34 | .494 | 1.166750 | 1.685745 | -2.259097 | 4.592597 |
|  | Equal variances not assumed |  |  | .692 | 32.260 | .494 | 1.166750 | 1.686105 | -2.266647 | 4.600147 |
| Planned opening distance (mm) | Equal variances assumed | 3.746 | .061 | 1.240 | 34 | .223 | 1.841750 | 1.484938 | -1.176007 | 4.859507 |
|  | Equal variances not assumed |  |  | 1.303 | 32.388 | .202 | 1.841750 | 1.413070 | -1.035229 | 4.718729 |
| Planned opening degree (°) | Equal variances assumed | .904 | .348 | 1.013 | 34 | .318 | 1.381125 | 1.363535 | -1.389911 | 4.152161 |
|  | Equal variances not assumed |  |  | 1.031 | 33.813 | .310 | 1.381125 | 1.339703 | -1.342034 | 4.104284 |
| Actual planned opening spread | Equal variances assumed | .897 | .350 | -1.075 | 34 | .290 | -1.121250 | 1.042557 | -3.239980 | .997480 |
|  | Equal variances not assumed |  |  | -1.125 | 32.952 | .269 | -1.121250 | .996759 | -3.149285 | .906785 |
| Actual planned opening degree spread（°） | Equal variances assumed | .084 | .774 | -.163 | 34 | .871 | -.214375 | 1.312774 | -2.882253 | 2.453503 |
|  | Equal variances not assumed |  |  | -.165 | 33.217 | .870 | -.214375 | 1.301327 | -2.861288 | 2.432538 |
| insall index | Equal variances assumed | 1.229 | .275 | -.775 | 34 | .444 | -.066975 | .086429 | -.242619 | .108669 |
|  | Equal variances not assumed |  |  | -.784 | 33.465 | .438 | -.066975 | .085413 | -.240658 | .106708 |
| pre-op WOMAC | Equal variances assumed | .277 | .602 | .732 | 34 | .469 | 3.613 | 4.933 | -6.414 | 13.639 |
|  | Equal variances not assumed |  |  | .750 | 33.992 | .458 | 3.613 | 4.816 | -6.175 | 13.400 |
| post-op WOMAC | Equal variances assumed | .858 | .361 | .497 | 34 | .622 | 1.700 | 3.421 | -5.253 | 8.653 |
|  | Equal variances not assumed |  |  | .527 | 31.009 | .602 | 1.700 | 3.227 | -4.881 | 8.281 |
| Pre- and post-operative WOMAC difference | Equal variances assumed | .803 | .376 | .797 | 34 | .431 | 1.913 | 2.400 | -2.964 | 6.789 |
|  | Equal variances not assumed |  |  | .766 | 25.819 | .451 | 1.913 | 2.497 | -3.222 | 7.047 |
| Postoperative lengthening of the affected limb | Equal variances assumed | 6.322 | .017 | -1.780 | 34 | .084 | -5.15000 | 2.89321 | -11.02971 | .72971 |
|  | Equal variances not assumed |  |  | -1.628 | 18.058 | .121 | -5.15000 | 3.16265 | -11.79294 | 1.49294 |
| Healing time（months） | Equal variances assumed | 1.904 | .177 | 3.365 | 34 | .002 | 1.2125 | .3603 | .4802 | 1.9448 |
|  | Equal variances not assumed |  |  | 3.707 | 22.876 | .001 | 1.2125 | .3271 | .5356 | 1.8894 |
| Pre-op MAD（mm） | Equal variances assumed | 4.572 | .040 | -1.105 | 34 | .277 | -7.314125 | 6.619173 | -20.765903 | 6.137653 |
|  | Equal variances not assumed |  |  | -1.180 | 29.763 | .247 | -7.314125 | 6.200052 | -19.980551 | 5.352301 |
| Post-op MAD（mm） | Equal variances assumed | .303 | .586 | -.129 | 34 | .898 | -.348125 | 2.705331 | -5.846019 | 5.149769 |
|  | Equal variances not assumed |  |  | -.129 | 32.809 | .898 | -.348125 | 2.693033 | -5.828357 | 5.132107 |
| Pre-op mLDFA （°） | Equal variances assumed | .000 | 1.000 | -1.741 | 34 | .091 | -1.647750 | .946237 | -3.570736 | .275236 |
|  | Equal variances not assumed |  |  | -1.757 | 33.249 | .088 | -1.647750 | .937640 | -3.554851 | .259351 |
| Post-op mLDFA （°） | Equal variances assumed | .001 | .982 | -.399 | 34 | .692 | -.452875 | 1.134727 | -2.758918 | 1.853168 |
|  | Equal variances not assumed |  |  | -.405 | 33.598 | .688 | -.452875 | 1.119262 | -2.728492 | 1.822742 |
| MAD correction (mm） | Equal variances assumed | 3.463 | .071 | 1.020 | 34 | .315 | 6.966000 | 6.827223 | -6.908586 | 20.840586 |
|  | Equal variances not assumed |  |  | 1.089 | 29.873 | .285 | 6.966000 | 6.398559 | -6.103931 | 20.035931 |
| mLDFA correction (°) | Equal variances assumed | .699 | .409 | .920 | 34 | .364 | 1.194875 | 1.298645 | -1.444289 | 3.834039 |
|  | Equal variances not assumed |  |  | .951 | 33.854 | .348 | 1.194875 | 1.256555 | -1.359160 | 3.748910 |
| WOMAC improvement ratio | Equal variances assumed | .727 | .400 | .252 | 34 | .803 | .014984 | .059523 | -.105981 | .135948 |
|  | Equal variances not assumed |  |  | .244 | 27.237 | .809 | .014984 | .061446 | -.111043 | .141010 |
| MAD correction ratio | Equal variances assumed | .007 | .935 | -.210 | 34 | .835 | -.022271 | .106120 | -.237932 | .193390 |
|  | Equal variances not assumed |  |  | -.208 | 31.387 | .836 | -.022271 | .106837 | -.240057 | .195516 |
| mLDFA correction ratio | Equal variances assumed | 1.060 | .311 | 1.038 | 34 | .307 | .017322 | .016689 | -.016594 | .051238 |
|  | Equal variances not assumed |  |  | 1.077 | 33.670 | .289 | .017322 | .016090 | -.015389 | .050034 |
| Height | Equal variances assumed | .218 | .643 | 1.279 | 34 | .209 | .03163 | .02472 | -.01861 | .08186 |
|  | Equal variances not assumed |  |  | 1.294 | 33.415 | .205 | .03163 | .02445 | -.01809 | .08134 |
| Body weight | Equal variances assumed | 2.102 | .156 | 1.887 | 34 | .068 | 4.57500 | 2.42462 | -.35242 | 9.50242 |
|  | Equal variances not assumed |  |  | 1.959 | 33.612 | .058 | 4.57500 | 2.33559 | -.17350 | 9.32350 |
| BMI | Equal variances assumed | .024 | .878 | 1.250 | 34 | .220 | .709574 | .567493 | -.443710 | 1.862857 |
|  | Equal variances not assumed |  |  | 1.256 | 32.807 | .218 | .709574 | .564922 | -.440026 | 1.859174 |

The time to intraoperative fracture healing occurred was 3.900 ± 1.392 months, while the time to postoperative fracture healing occurred was 2.688 ± 0.403 months, p=0.002

| **group statistic** | | | | | |
| --- | --- | --- | --- | --- | --- |
|  | PHF type | N | mean | S.E. | Standard error of the mean |
| age | 1.00 | 8 | 61.13 | 11.294 | 3.993 |
|  | 2.00 | 14 | 58.21 | 8.229 | 2.199 |
| Actual opening distance (mm) | 1.00 | 8 | 14.46000 | 5.588685 | 1.975899 |
|  | 2.00 | 14 | 11.63714 | 3.692953 | .986983 |
| Actual opening degree(°) | 1.00 | 8 | 14.22625 | 5.806996 | 2.053083 |
|  | 2.00 | 14 | 11.28743 | 4.217704 | 1.127229 |
| Planned opening distance (mm) | 1.00 | 8 | 12.84500 | 5.909124 | 2.089191 |
|  | 2.00 | 14 | 10.30857 | 4.403221 | 1.176810 |
| Planned opening degree (°) | 1.00 | 8 | 12.03250 | 5.035913 | 1.780464 |
|  | 2.00 | 14 | 9.72000 | 3.649105 | .975264 |
| Actual planned opening spread | 1.00 | 8 | 1.61500 | 3.589592 | 1.269112 |
|  | 2.00 | 14 | 1.32857 | 3.462515 | .925396 |
| Actual planned opening degree spread（°） | 1.00 | 8 | 2.19375 | 5.785452 | 2.045466 |
|  | 2.00 | 14 | 1.56743 | 2.426045 | .648388 |
| insall index | 1.00 | 8 | 1.25913 | .255007 | .090159 |
|  | 2.00 | 14 | 1.14850 | .267346 | .071451 |
| pre-op WOMAC | 1.00 | 8 | 28.25 | 14.945 | 5.284 |
|  | 2.00 | 14 | 27.14 | 16.524 | 4.416 |
| post-op WOMAC | 1.00 | 8 | 11.88 | 12.276 | 4.340 |
|  | 2.00 | 14 | 10.50 | 11.785 | 3.150 |
| Pre- and post-operative WOMAC difference | 1.00 | 8 | 16.38 | 5.655 | 1.999 |
|  | 2.00 | 14 | 16.64 | 6.344 | 1.696 |
| Postoperative lengthening of the affected limb | 1.00 | 8 | 4.8750 | 5.54044 | 1.95884 |
|  | 2.00 | 14 | 3.0000 | 3.50823 | .93761 |
| Healing time（months） | 1.00 | 8 | 4.500 | 2.1381 | .7559 |
|  | 2.00 | 14 | 3.357 | .3631 | .0971 |
| Pre-op MAD（mm） | 1.00 | 8 | -42.94000 | 28.985140 | 10.247794 |
|  | 2.00 | 14 | -32.49643 | 19.322968 | 5.164280 |
| Post-op MAD（mm） | 1.00 | 8 | 2.31000 | 7.122706 | 2.518257 |
|  | 2.00 | 14 | -1.23071 | 8.622941 | 2.304578 |
| Pre-op mLDFA （°） | 1.00 | 8 | 81.08875 | 3.127306 | 1.105670 |
|  | 2.00 | 14 | 83.36857 | 2.414733 | .645365 |
| Post-op mLDFA （°） | 1.00 | 8 | 92.38000 | 4.556101 | 1.610825 |
|  | 2.00 | 14 | 93.31000 | 2.820941 | .753928 |
| MAD correction (mm） | 1.00 | 8 | 45.25000 | 31.970030 | 11.303113 |
|  | 2.00 | 14 | 31.26571 | 18.196218 | 4.863144 |
| mLDFA correction (°) | 1.00 | 8 | 11.29125 | 4.274206 | 1.511160 |
|  | 2.00 | 14 | 9.94143 | 4.374264 | 1.169071 |
| WOMAC improvement ratio | 1.00 | 8 | .64934 | .198945 | .070338 |
|  | 2.00 | 14 | .65527 | .114823 | .030688 |
| MAD correction ratio | 1.00 | 8 | 1.04086 | .275510 | .097407 |
|  | 2.00 | 14 | .97800 | .348247 | .093073 |
| mLDFA correction ratio | 1.00 | 8 | .13991 | .053454 | .018899 |
|  | 2.00 | 14 | .12053 | .057397 | .015340 |
| Height | 1.00 | 8 | 1.6500 | .07764 | .02745 |
|  | 2.00 | 14 | 1.6807 | .07227 | .01931 |
| Body weight | 1.00 | 8 | 65.3750 | 6.13974 | 2.17073 |
|  | 2.00 | 14 | 66.6429 | 8.84575 | 2.36413 |
| BMI | 1.00 | 8 | 24.02270 | 1.817088 | .642438 |
|  | 2.00 | 14 | 23.48486 | 1.583627 | .423242 |

1: type 1 fracture

2: type 3 fracture

| **independent sample test** | | | | | | | | | | |
| --- | --- | --- | --- | --- | --- | --- | --- | --- | --- | --- |
|  | | Levene’s Test for Equality of Variances | | t-test for Equality of Means | | | | | | |
|  |  | F | Sig. | t | df | Sig.(2-tailed) | Mean Difference | standard error value | 95% Confidence Interval of the Difference | |
|  |  |  |  |  |  |  |  |  | Lower | Upper |
| age | Equal variances assumed | .605 | .446 | .697 | 20 | .494 | 2.911 | 4.173 | -5.794 | 11.616 |
|  | Equal variances not assumed |  |  | .639 | 11.330 | .536 | 2.911 | 4.559 | -7.087 | 12.909 |
| Actual opening distance (mm) | Equal variances assumed | .026 | .873 | 1.432 | 20 | .168 | 2.822857 | 1.971944 | -1.290546 | 6.936261 |
|  | Equal variances not assumed |  |  | 1.278 | 10.574 | .229 | 2.822857 | 2.208690 | -2.062411 | 7.708125 |
| Actual opening degree(°) | Equal variances assumed | .806 | .380 | 1.372 | 20 | .185 | 2.938821 | 2.142338 | -1.530016 | 7.407659 |
|  | Equal variances not assumed |  |  | 1.255 | 11.303 | .235 | 2.938821 | 2.342177 | -2.199451 | 8.077093 |
| Planned opening distance (mm) | Equal variances assumed | .957 | .340 | 1.149 | 20 | .264 | 2.536429 | 2.208183 | -2.069760 | 7.142617 |
|  | Equal variances not assumed |  |  | 1.058 | 11.522 | .312 | 2.536429 | 2.397832 | -2.712130 | 7.784987 |
| Planned opening degree (°) | Equal variances assumed | 2.481 | .131 | 1.246 | 20 | .227 | 2.312500 | 1.855719 | -1.558462 | 6.183462 |
|  | Equal variances not assumed |  |  | 1.139 | 11.284 | .278 | 2.312500 | 2.030072 | -2.141989 | 6.766989 |
| Actual planned opening spread | Equal variances assumed | .466 | .502 | .184 | 20 | .856 | .286429 | 1.554540 | -2.956286 | 3.529143 |
|  | Equal variances not assumed |  |  | .182 | 14.253 | .858 | .286429 | 1.570670 | -3.076725 | 3.649582 |
| Actual planned opening degree spread（°） | Equal variances assumed | 10.175 | .005 | .358 | 20 | .724 | .626321 | 1.747181 | -3.018233 | 4.270876 |
|  | Equal variances not assumed |  |  | .292 | 8.432 | .777 | .626321 | 2.145772 | -4.278097 | 5.530740 |
| insall index | Equal variances assumed | .001 | .972 | .949 | 20 | .354 | .110625 | .116604 | -.132606 | .353856 |
|  | Equal variances not assumed |  |  | .962 | 15.304 | .351 | .110625 | .115039 | -.134151 | .355401 |
| pre-op WOMAC | Equal variances assumed | .039 | .846 | .156 | 20 | .877 | 1.107 | 7.087 | -13.675 | 15.889 |
|  | Equal variances not assumed |  |  | .161 | 15.993 | .874 | 1.107 | 6.886 | -13.492 | 15.706 |
| post-op WOMAC | Equal variances assumed | .223 | .642 | .259 | 20 | .798 | 1.375 | 5.300 | -9.681 | 12.431 |
|  | Equal variances not assumed |  |  | .256 | 14.195 | .801 | 1.375 | 5.363 | -10.112 | 12.862 |
| Pre- and post-operative WOMAC difference | Equal variances assumed | .211 | .651 | -.099 | 20 | .922 | -.268 | 2.709 | -5.918 | 5.383 |
|  | Equal variances not assumed |  |  | -.102 | 16.182 | .920 | -.268 | 2.622 | -5.820 | 5.285 |
| Postoperative lengthening of the affected limb | Equal variances assumed | 5.884 | .025 | .977 | 20 | .340 | 1.87500 | 1.91880 | -2.12755 | 5.87755 |
|  | Equal variances not assumed |  |  | .863 | 10.284 | .408 | 1.87500 | 2.17168 | -2.94572 | 6.69572 |
| Healing time（months） | Equal variances assumed | 10.783 | .004 | 1.986 | 20 | .061 | 1.1429 | .5754 | -.0575 | 2.3432 |
|  | Equal variances not assumed |  |  | 1.500 | 7.232 | .176 | 1.1429 | .7621 | -.6477 | 2.9334 |
| Pre-op MAD（mm） | Equal variances assumed | .799 | .382 | -1.017 | 20 | .321 | -10.443571 | 10.267997 | -31.862238 | 10.975095 |
|  | Equal variances not assumed |  |  | -.910 | 10.637 | .383 | -10.443571 | 11.475499 | -35.806405 | 14.919262 |
| Post-op MAD（mm） | Equal variances assumed | 3.150 | .091 | .983 | 20 | .337 | 3.540714 | 3.602977 | -3.974964 | 11.056393 |
|  | Equal variances not assumed |  |  | 1.037 | 17.156 | .314 | 3.540714 | 3.413605 | -3.656392 | 10.737821 |
| Pre-op mLDFA （°） | Equal variances assumed | .289 | .597 | -1.915 | 20 | .070 | -2.279821 | 1.190321 | -4.762787 | .203144 |
|  | Equal variances not assumed |  |  | -1.781 | 11.842 | .101 | -2.279821 | 1.280235 | -5.073345 | .513703 |
| Post-op mLDFA （°） | Equal variances assumed | 2.105 | .162 | -.595 | 20 | .559 | -.930000 | 1.563056 | -4.190478 | 2.330478 |
|  | Equal variances not assumed |  |  | -.523 | 10.141 | .612 | -.930000 | 1.778529 | -4.885368 | 3.025368 |
| MAD correction (mm） | Equal variances assumed | 1.498 | .235 | 1.318 | 20 | .202 | 13.984286 | 10.608623 | -8.144913 | 36.113484 |
|  | Equal variances not assumed |  |  | 1.136 | 9.653 | .283 | 13.984286 | 12.304898 | -13.566781 | 41.535352 |
| mLDFA correction (°) | Equal variances assumed | .020 | .889 | .702 | 20 | .491 | 1.349821 | 1.923281 | -2.662071 | 5.361714 |
|  | Equal variances not assumed |  |  | .706 | 14.994 | .491 | 1.349821 | 1.910584 | -2.722628 | 5.422271 |
| WOMAC improvement ratio | Equal variances assumed | 1.218 | .283 | -.089 | 20 | .930 | -.005926 | .066366 | -.144362 | .132511 |
|  | Equal variances not assumed |  |  | -.077 | 9.729 | .940 | -.005926 | .076741 | -.177563 | .165712 |
| MAD correction ratio | Equal variances assumed | 2.240 | .150 | .437 | 20 | .667 | .062868 | .143885 | -.237271 | .363007 |
|  | Equal variances not assumed |  |  | .467 | 17.681 | .646 | .062868 | .134725 | -.220545 | .346281 |
| mLDFA correction ratio | Equal variances assumed | .006 | .937 | .780 | 20 | .445 | .019377 | .024841 | -.032441 | .071194 |
|  | Equal variances not assumed |  |  | .796 | 15.613 | .438 | .019377 | .024341 | -.032328 | .071082 |
| Height | Equal variances assumed | .071 | .793 | -.934 | 20 | .361 | -.03071 | .03288 | -.09931 | .03788 |
|  | Equal variances not assumed |  |  | -.915 | 13.822 | .376 | -.03071 | .03356 | -.10279 | .04136 |
| Body weight | Equal variances assumed | 2.298 | .145 | -.357 | 20 | .725 | -1.26786 | 3.54713 | -8.66704 | 6.13133 |
|  | Equal variances not assumed |  |  | -.395 | 19.034 | .697 | -1.26786 | 3.20954 | -7.98468 | 5.44897 |
| BMI | Equal variances assumed | .079 | .782 | .727 | 20 | .476 | .537842 | .739730 | -1.005209 | 2.080893 |
|  | Equal variances not assumed |  |  | .699 | 13.069 | .497 | .537842 | .769324 | -1.123287 | 2.198971 |

| **group statistic** | | | | | |
| --- | --- | --- | --- | --- | --- |
|  | PHF type | N | mean | S.E. | mean的标准误 |
| age | 3.00 | 10 | 60.20 | 11.063 | 3.499 |
|  | 4.00 | 4 | 60.25 | 13.865 | 6.933 |
| Actual opening distance (mm) | 3.00 | 10 | 12.68900 | 2.605168 | .823826 |
|  | 4.00 | 4 | 12.41250 | 3.037986 | 1.518993 |
| Actual opening degree(°) | 3.00 | 10 | 10.81800 | 5.738193 | 1.814576 |
|  | 4.00 | 4 | 14.01750 | 3.201139 | 1.600570 |
| Planned opening distance (mm) | 3.00 | 10 | 9.96100 | 3.208196 | 1.014521 |
|  | 4.00 | 4 | 9.01500 | 4.157471 | 2.078736 |
| Planned opening degree (°) | 3.00 | 10 | 9.60200 | 3.898766 | 1.232898 |
|  | 4.00 | 4 | 9.00500 | 4.173891 | 2.086946 |
| Actual planned opening spread | 3.00 | 10 | 2.72800 | 2.562931 | .810470 |
|  | 4.00 | 4 | 3.39750 | 2.151904 | 1.075952 |
| Actual planned opening degree spread（°） | 3.00 | 10 | 1.21600 | 3.468551 | 1.096852 |
|  | 4.00 | 4 | 5.01250 | 4.351033 | 2.175517 |
| insall index | 3.00 | 10 | 1.28330 | .244391 | .077283 |
|  | 4.00 | 4 | 1.27350 | .283375 | .141688 |
| pre-op WOMAC | 3.00 | 10 | 23.10 | 11.609 | 3.671 |
|  | 4.00 | 4 | 33.25 | 16.581 | 8.290 |
| post-op WOMAC | 3.00 | 10 | 8.40 | 7.137 | 2.257 |
|  | 4.00 | 4 | 14.75 | 6.185 | 3.092 |
| Pre- and post-operative WOMAC difference | 3.00 | 10 | 14.70 | 6.848 | 2.166 |
|  | 4.00 | 4 | 18.50 | 13.626 | 6.813 |
| Postoperative lengthening of the affected limb | 3.00 | 10 | 11.2000 | 13.77437 | 4.35584 |
|  | 4.00 | 4 | 4.7500 | 9.50000 | 4.75000 |
| Healing time（months） | 3.00 | 10 | 2.800 | .4830 | .1528 |
|  | 4.00 | 4 | 2.500 | .0000 | .0000 |
| Pre-op MAD（mm） | 3.00 | 10 | -31.15500 | 13.775416 | 4.356169 |
|  | 4.00 | 4 | -30.50000 | 12.583057 | 6.291529 |
| Post-op MAD（mm） | 3.00 | 10 | .63600 | 8.470042 | 2.678462 |
|  | 4.00 | 4 | 4.55250 | 6.143915 | 3.071958 |
| Pre-op mLDFA （°） | 3.00 | 10 | 83.45600 | 2.957169 | .935139 |
|  | 4.00 | 4 | 85.07500 | 2.680019 | 1.340009 |
| Post-op mLDFA （°） | 3.00 | 10 | 92.35500 | 2.756315 | .871623 |
|  | 4.00 | 4 | 96.75000 | 1.892969 | .946485 |
| MAD correction (mm） | 3.00 | 10 | 31.79100 | 12.412790 | 3.925269 |
|  | 4.00 | 4 | 35.05250 | 13.341827 | 6.670914 |
| mLDFA correction (°) | 3.00 | 10 | 8.89900 | 3.044640 | .962800 |
|  | 4.00 | 4 | 11.67500 | 2.963528 | 1.481764 |
| WOMAC improvement ratio | 3.00 | 10 | .68832 | .216572 | .068486 |
|  | 4.00 | 4 | .52969 | .207544 | .103772 |
| MAD correction ratio | 3.00 | 10 | 1.06468 | .315729 | .099842 |
|  | 4.00 | 4 | 1.18134 | .296076 | .148038 |
| mLDFA correction ratio | 3.00 | 10 | .10743 | .039014 | .012337 |
|  | 4.00 | 4 | .13799 | .039239 | .019619 |
| Height | 3.00 | 10 | 1.6370 | .07889 | .02495 |
|  | 4.00 | 4 | 1.6375 | .06752 | .03376 |
| Body weight | 3.00 | 10 | 61.5000 | 6.68747 | 2.11476 |
|  | 4.00 | 4 | 62.0000 | 5.59762 | 2.79881 |
| BMI | 3.00 | 10 | 22.93442 | 1.785133 | .564509 |
|  | 4.00 | 4 | 23.14736 | 1.982538 | .991269 |

3: type 1 fracture

4: type 3 fracture

| **independent sample test** | | | | | | | | | | |
| --- | --- | --- | --- | --- | --- | --- | --- | --- | --- | --- |
|  | | Levene’s Test for Equality of Variances | | t-test for Equality of Means | | | | | | |
|  |  | F | Sig. | t | df | Sig.(2-tailed) | Mean Difference | standard error value | 95% Confidence Interval of the Difference | |
|  |  |  |  |  |  |  |  |  | Lower | Upper |
| age | Equal variances assumed | .363 | .558 | -.007 | 12 | .994 | -.050 | 6.997 | -15.294 | 15.194 |
|  | Equal variances not assumed |  |  | -.006 | 4.623 | .995 | -.050 | 7.765 | -20.512 | 20.412 |
| Actual opening distance (mm) | Equal variances assumed | .396 | .541 | .172 | 12 | .866 | .276500 | 1.609077 | -3.229378 | 3.782378 |
|  | Equal variances not assumed |  |  | .160 | 4.884 | .879 | .276500 | 1.728013 | -4.197539 | 4.750539 |
| Actual opening degree(°) | Equal variances assumed | 1.768 | .208 | -1.036 | 12 | .321 | -3.199500 | 3.088679 | -9.929154 | 3.530154 |
|  | Equal variances not assumed |  |  | -1.322 | 10.104 | .215 | -3.199500 | 2.419609 | -8.583222 | 2.184222 |
| Planned opening distance (mm) | Equal variances assumed | .538 | .478 | .461 | 12 | .653 | .946000 | 2.052848 | -3.526772 | 5.418772 |
|  | Equal variances not assumed |  |  | .409 | 4.514 | .701 | .946000 | 2.313092 | -5.197811 | 7.089811 |
| Planned opening degree (°) | Equal variances assumed | .004 | .950 | .254 | 12 | .804 | .597000 | 2.348290 | -4.519485 | 5.713485 |
|  | Equal variances not assumed |  |  | .246 | 5.246 | .815 | .597000 | 2.423918 | -5.546871 | 6.740871 |
| Actual planned opening spread | Equal variances assumed | .304 | .591 | -.459 | 12 | .655 | -.669500 | 1.459263 | -3.848960 | 2.509960 |
|  | Equal variances not assumed |  |  | -.497 | 6.656 | .635 | -.669500 | 1.347047 | -3.888443 | 2.549443 |
| Actual planned opening degree spread（°） | Equal variances assumed | .063 | .806 | -1.730 | 12 | .109 | -3.796500 | 2.194220 | -8.577296 | .984296 |
|  | Equal variances not assumed |  |  | -1.558 | 4.620 | .185 | -3.796500 | 2.436382 | -10.217791 | 2.624791 |
| insall index | Equal variances assumed | .088 | .772 | .065 | 12 | .949 | .009800 | .150681 | -.318505 | .338105 |
|  | Equal variances not assumed |  |  | .061 | 4.906 | .954 | .009800 | .161394 | -.407483 | .427083 |
| pre-op WOMAC | Equal variances assumed | .507 | .490 | -1.317 | 12 | .213 | -10.150 | 7.709 | -26.947 | 6.647 |
|  | Equal variances not assumed |  |  | -1.119 | 4.238 | .322 | -10.150 | 9.067 | -34.776 | 14.476 |
| post-op WOMAC | Equal variances assumed | .023 | .881 | -1.553 | 12 | .146 | -6.350 | 4.089 | -15.258 | 2.558 |
|  | Equal variances not assumed |  |  | -1.659 | 6.438 | .145 | -6.350 | 3.828 | -15.565 | 2.865 |
| Pre- and post-operative WOMAC difference | Equal variances assumed | 2.121 | .171 | -.711 | 12 | .491 | -3.800 | 5.344 | -15.443 | 7.843 |
|  | Equal variances not assumed |  |  | -.532 | 3.625 | .626 | -3.800 | 7.149 | -24.486 | 16.886 |
| Postoperative lengthening of the affected limb | Equal variances assumed | .339 | .571 | .849 | 12 | .412 | 6.45000 | 7.59618 | -10.10064 | 23.00064 |
|  | Equal variances not assumed |  |  | 1.001 | 8.228 | .345 | 6.45000 | 6.44483 | -8.34054 | 21.24054 |
| Healing time（months） | Equal variances assumed | 18.000 | .001 | 1.212 | 12 | .249 | .3000 | .2475 | -.2392 | .8392 |
|  | Equal variances not assumed |  |  | 1.964 | 9.000 | .081 | .3000 | .1528 | -.0456 | .6456 |
| Pre-op MAD（mm） | Equal variances assumed | .083 | .778 | -.082 | 12 | .936 | -.655000 | 7.979142 | -18.040057 | 16.730057 |
|  | Equal variances not assumed |  |  | -.086 | 6.099 | .935 | -.655000 | 7.652421 | -19.306616 | 17.996616 |
| Post-op MAD（mm） | Equal variances assumed | 1.473 | .248 | -.832 | 12 | .421 | -3.916500 | 4.704795 | -14.167367 | 6.334367 |
|  | Equal variances not assumed |  |  | -.961 | 7.794 | .365 | -3.916500 | 4.075670 | -13.358487 | 5.525487 |
| Pre-op mLDFA （°） | Equal variances assumed | .479 | .502 | -.947 | 12 | .362 | -1.619000 | 1.709968 | -5.344701 | 2.106701 |
|  | Equal variances not assumed |  |  | -.991 | 6.148 | .359 | -1.619000 | 1.634047 | -5.594219 | 2.356219 |
| Post-op mLDFA （°） | Equal variances assumed | 1.276 | .281 | -2.893 | 12 | .013 | -4.395000 | 1.519153 | -7.704950 | -1.085050 |
|  | Equal variances not assumed |  |  | -3.416 | 8.265 | .009 | -4.395000 | 1.286686 | -7.345644 | -1.444356 |
| MAD correction (mm） | Equal variances assumed | .221 | .647 | -.436 | 12 | .671 | -3.261500 | 7.484697 | -19.569253 | 13.046253 |
|  | Equal variances not assumed |  |  | -.421 | 5.228 | .690 | -3.261500 | 7.740079 | -22.899704 | 16.376704 |
| mLDFA correction (°) | Equal variances assumed | .076 | .788 | -1.551 | 12 | .147 | -2.776000 | 1.789357 | -6.674675 | 1.122675 |
|  | Equal variances not assumed |  |  | -1.571 | 5.728 | .170 | -2.776000 | 1.767090 | -7.150254 | 1.598254 |
| WOMAC improvement ratio | Equal variances assumed | .041 | .842 | 1.251 | 12 | .235 | .158632 | .126812 | -.117667 | .434930 |
|  | Equal variances not assumed |  |  | 1.276 | 5.815 | .251 | .158632 | .124334 | -.147967 | .465231 |
| MAD correction ratio | Equal variances assumed | .396 | .541 | -.634 | 12 | .538 | -.116659 | .183950 | -.517451 | .284133 |
|  | Equal variances not assumed |  |  | -.653 | 5.940 | .538 | -.116659 | .178560 | -.554648 | .321330 |
| mLDFA correction ratio | Equal variances assumed | .035 | .854 | -1.322 | 12 | .211 | -.030554 | .023114 | -.080916 | .019808 |
|  | Equal variances not assumed |  |  | -1.318 | 5.552 | .239 | -.030554 | .023176 | -.088391 | .027283 |
| Height | Equal variances assumed | .115 | .740 | -.011 | 12 | .991 | -.00050 | .04508 | -.09873 | .09773 |
|  | Equal variances not assumed |  |  | -.012 | 6.523 | .991 | -.00050 | .04198 | -.10125 | .10025 |
| Body weight | Equal variances assumed | .477 | .503 | -.131 | 12 | .898 | -.50000 | 3.80542 | -8.79131 | 7.79131 |
|  | Equal variances not assumed |  |  | -.143 | 6.678 | .891 | -.50000 | 3.50793 | -8.87675 | 7.87675 |
| BMI | Equal variances assumed | .420 | .529 | -.196 | 12 | .848 | -.212936 | 1.086473 | -2.580157 | 2.154286 |
|  | Equal variances not assumed |  |  | -.187 | 5.083 | .859 | -.212936 | 1.140738 | -3.130925 | 2.705053 |

Postoperative fracture type correlated with mPost-op mLDFA (°), and type 3 postoperative fractures had larger postoperative mLDFA values than type 1 postoperative fractures, with type I postoperative fracture having a postoperative mLDFA value of 92.355±2.756 compared with type 3 postoperative fracture having a postoperative mLDFA value of 96.750±1.893. In other words, overcorrection of the mLDFA value can cause a type III postoperative fractures.

|  | | | | |
| --- | --- | --- | --- | --- |
|  | | | | |
|  | | Intraoperative fracture classifcation | | total |
|  |  | Type 1 | Type 3 |  |
| Laterality | Right | 4 | 10 | 14 |
|  | Left | 5 | 1 | 6 |
| total | | 9 | 11 | 20 |

p=0.024 Patients with intraoperative fractures have an increased risk of type III fractures in the right knee.

|  | | | | |
| --- | --- | --- | --- | --- |
|  | | | | |
|  | | fracture or no | | count |
|  |  | 0 | 1 |  |
| sex | 1 | 3 | 8 | 11 |
|  | 2 | 49 | 27 | 76 |
| count | | 52 | 35 | 87 |

p=0.019 OR=0.0207 95% CI(0.051~0.084)
